# The role of miR-128 in cancer development, prevention, drug resistance, and immunotherapy

**DOI:** 10.3389/fonc.2022.1067974

**Published:** 2023-01-19

**Authors:** Hendrik Setia Budi, Laith A. Younus, Methaq Hadi Lafta, Sameena Parveen, Hawraa Jabbar Mohammad, Zahraa Haleem Al-qaim, Mohammed Abed Jawad, Rosario Mireya Romero Parra, Yasser Fakri Mustafa, Firas Rahi Alhachami, Sajad Karampoor, Rasoul Mirzaei

**Affiliations:** ^1^ Department of Oral Biology, Dental Pharmacology, Faculty of Dental Medicine, Universitas Airlangga, Surabaya, Indonesia; ^2^ Department of Clinical Laboratory Sciences, Faculty of Pharmacy, Jabir Ibn, Hayyan Medical University, Al Najaf Al Ashraf, Iraq; ^3^ Iraqi Ministry of Education, Baghdad, Iraq; ^4^ Department of Maxillofacial Surgery and Diagnostic Sciences, College of Dentistry, Jazan University, Jazan, Saudi Arabia; ^5^ Al-Manara College For Medical Sciences, Maysan, Iraq; ^6^ Department of Anesthesia Techniques , Al-Mustaqbal University College, Hilla, Iraq; ^7^ Al-Nisour University College, Baghdad, Iraq; ^8^ Universidad Continental, Lima, Peru; ^9^ Department of Pharmaceutical Chemistry, College of Pharmacy, University of Mosul, Mosul, Iraq; ^10^ Radiology Department, College of Health and Medical Technology, Al-Ayen University, Thi-Qar, Nasiriyah, Iraq; ^11^ Gastrointestinal and Liver Diseases Research Center, Iran University of Medical Sciences, Tehran, Iran; ^12^ Venom and Biotherapeutics Molecules Lab, Medical Biotechnology Department, Biotechnology Research Center, Pasteur Institute of Iran, Tehran, Iran

**Keywords:** miR-128, cancer progression, cancer suppression, chemoresistance, immunotherapy

## Abstract

A growing body of evidence has revealed that microRNA (miRNA) expression is dysregulated in cancer, and they can act as either oncogenes or suppressors under certain conditions. Furthermore, some studies have discovered that miRNAs play a role in cancer cell drug resistance by targeting drug-resistance-related genes or influencing genes involved in cell proliferation, cell cycle, and apoptosis. In this regard, the abnormal expression of miRNA-128 (miR-128) has been found in various human malignancies, and its verified target genes are essential in cancer-related processes, including apoptosis, cell propagation, and differentiation. This review will discuss the functions and processes of miR-128 in multiple cancer types. Furthermore, the possible involvement of miR-128 in cancer drug resistance and tumor immunotherapeutic will be addressed.

## 1 Introduction

Cancer is a serious threat to humanity that has recently overtaken heart disease as the leading cause of human death (1). According to reports, millions of new cases were diagnosed worldwide in 2019, with an estimated 8.2 million cancer deaths (1). Cancer is becoming more common as people live longer and the global ecology deteriorates, so the incidence rate is expected to reach 23.6 million by 2030 (1). Cancer is a complex genetic disease in which oncogenic and/or suppressor gene mutations lead to impaired cell growth and death (2). In this sense, data show that microRNAs (miRNAs) influence the cause of human cancer (3–5). miRNAs are small non-coding RNAs of 18-24 nucleotides that exert functions such as mRNA degradation and inhibiting translation initiation. Also, various studies have shown the importance of miRNAs in controlling key cell functions such as apoptosis, growth, migration, proliferation, stress response, and metabolism. (6–8). miRNAs have also been shown to play an important role in the progression of diseases such as cancer. Dysregulation of miRNAs has also been shown in diseases such as cancer through various processes, including amplification or deletion of miRNA genes, inappropriate transcriptional regulation of miRNAs, and problems in the miRNA biosynthesis machinery (9–11). In this regard, a growing body of evidence shows that miRNA-128 (miR-128) is a well-known tumor suppressor, inhibiting cancer growth, migration and metastasis by upregulating cancer (12–14). miR-128 is an intronic miRNA, and the mature miR-128 form is encoded by the two isoforms, namely miR-128-1 and miR-128-2 (15). The pri-miR-128-1 gene is located in the R3H domain-containing protein 1 gene (R3HDM1) on chromosome 2q21.3. It has been shown that pri-miR-128-2 is located within the cAMP-regulated phosphoprotein, 21 kDa gene (ARPP21) on chromosome 3p22.3 (15). miR-128 is one of the most prevalent miRNAs expressed in the adult mouse and human brain and is tissue-dependent (16). In mice, miR-128 expression gradually rises during development and reaches a maximum in adulthood. Additionally, miR-128 is expressed in various brain areas, indicating a crucial involvement in the operation of different neuronal cells (16). Indeed, miR-128 has been shown to play a significant function in nervous system development and maintenance (17). miR-128 has modulated neuronal excitability and motor activity by decreasing the expression of different ion channels and extracellular signal-regulated kinase 2 (ERK2) signaling pathway components (16). In addition to its physiological roles in normal tissues, miR-128 plays an important regulatory role in tumor cells. Preliminary studies on miR-128 point to its tumor suppressor activity. Loss of miR-128 has been reported in human lung cancer—due to a deletion on chromosome 3p that includes the miR-128-2 and ARPP21 locus—and in breast cancer (18). Specifically, Kotani et al. found that miR-128 is downregulated in acute lymphocytic leukemia (ALL)-AF4 (19). Furthermore, one study showed reduced expression of miR-128 in chemoresistant breast cancer (BC) cells nourished from the BC cell line and primary BC, which was inserted before regulation involved in region 1 (Bmi-1) and ABC transporter of mouse B lymphoma 5 (ABCC5), are known as targets of miR-128 (20, 21). Additionally, miR-128 inhibits the p38 Mitogen-activated protein kinase (MAPK) signaling pathway, which reduces the production of interleukin (IL) 10 (IL-10) and IL-6 and, on the other hand, increases the formation of IL-12 in dendritic cells (DCs) and enhances DC antitumor immunity and the progression Reduces cancer in melanoma (22). Most importantly, Zhu and colleagues found that overexpression of miR-128 in the setting of doxorubicin lowers cell viability while increasing apoptosis and DNA damage, rendering BC-initiating cells more sensitive to therapy (23). They additionally discovered that decreased amounts of miR-128 in metastatic BC tissues were associated with poor clinical therapeutic efficacy and survival rates. The functions and processes of miR-128 in various types of cancer, such as BC, lung, glioblastoma, pancreatic, thyroid, osteosarcoma, leukemia, multiple myeloma, melanoma, and head and neck carcinoma, will be explored and described in this report. In addition, a possible function of miR-128 in cancer resistance to chemotherapeutics, as well as cancer immunotherapeutics in certain types of cancer, will be discussed.

## 2 Physiological and pathological functions of miR-128

**Figure 1:**
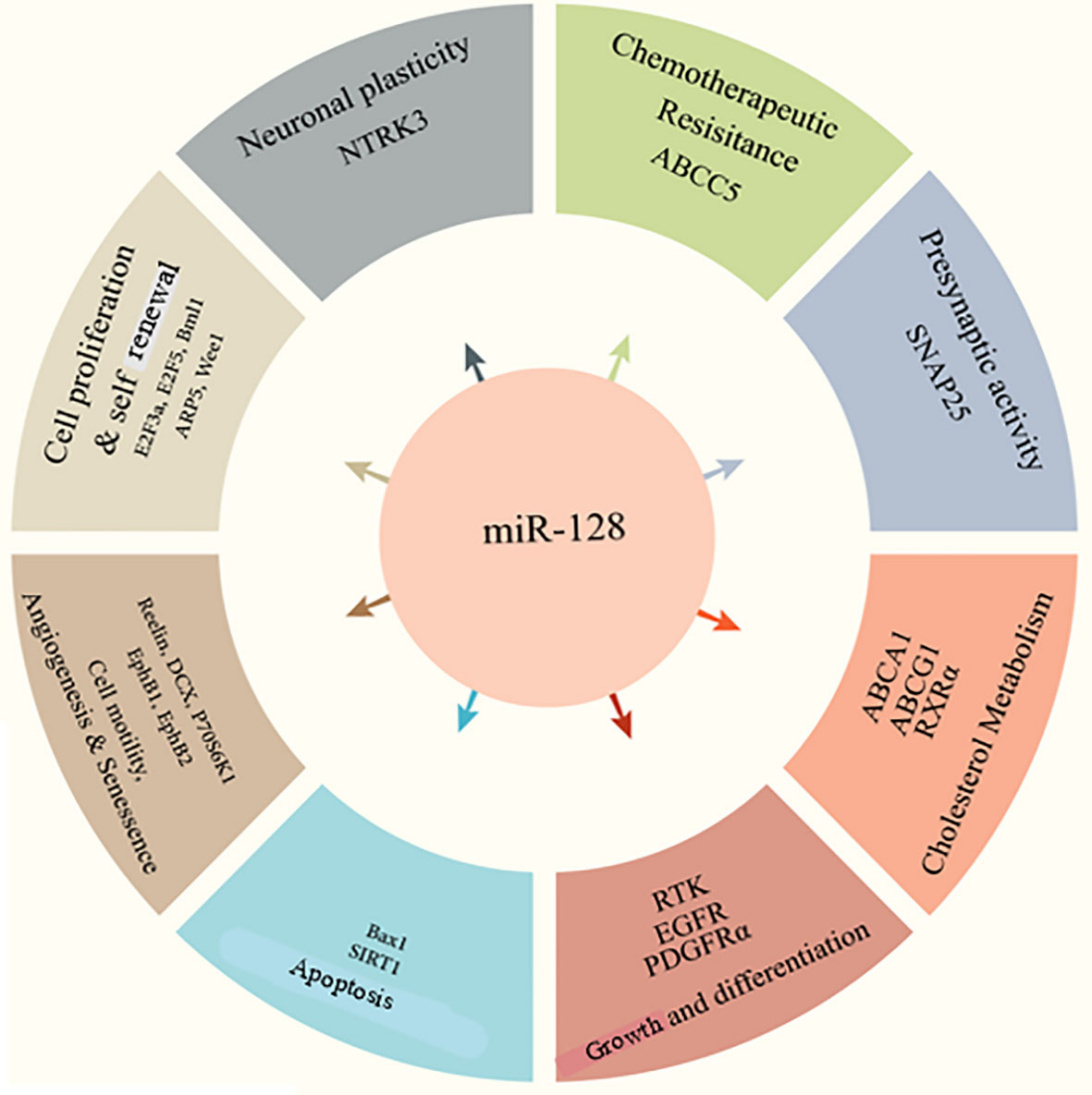
miR-128 functions in the different cellular processes.

miR-128 has been implicated in various diseases and cell processes, including cell division, epithelial-mesenchymal transition (EMT), tumor growth, angiogenesis, and invasion (**Figure 1**) (24–27). Of note, accumulating data suggest that miR-128 can be used as a prognostic indicator in various disorders (27–29). miR-128 upregulation increases neuronal development in embryonic neural stem cells and P19 cells primary by suppressing non-sense-mediated decaying (30, 31). Human-induced pluripotent stem cells transduced with miR-128 exhibit features comparable to mature neurons and increase the production of beta-tubulin as well as other neuronal indicators (32). Throughout embryonic mouse neurodevelopment, the brain-enriched miR-128 is plentiful and elevated (33). miR-128 was initially hypothesized as a physiological modulator of mRNA usage, similar to miR-124 (33). In a cell culture system, miR-128 was demonstrated to enhance neurogenesis by inhibiting the production of two proteins involved in nonsense-mediated mRNA degradation (NMD) (30). Different roles of miR-128 in cognition and memory were revealed later. Upregulation of miR-128 was relevant and required for the extinction of conditioned fear in research on the acquisition and inhibition of fearful memories (34).

Besides, miR-128 is vital in muscle renewal, revascularization, adipogenesis, and osteoclastogenesis (35). Recent research has shown that miR-128, a muscle-related miRNA, may limit cardiomyocyte migrations, propagation, and rejuvenation and control chicken myocardial inflammatory response (35–37). Additionally, miR-128 was found to be important in the tumorigenesis of skeletal muscle satellite cells (SMSCs) in *in vitro* experiments (35). In fact, miR-128 increased myogenic markers (myosin heavy chain (MHC), myocyte enhancer factor 2C (MEF2C), and myogenic differentiation (MyoD) in C2C12 cells *via* negatively impacting the Jun N-terminal kinase (JNK)/MAPK axis (38). In contrast, miR-128 suppression reduced SMSC maturation into myotubes at 2 and 3 days (39).

miR-128a has also been shown to influence cell growth, and it might play a role in adipogenesis and adipose tissue formation (40–42). However, miR-128-3p (a member of the miR-128 family) has not yet been associated with preadipocyte development or lipogenesis. Recently, the SERTA domain containing 2 (Sertad2) has been shown to modulate lipid metabolism, and peroxisome proliferator-activated receptor gamma (PPARγ) is a known key regulator of preadipocyte development (43). Furthermore, bioinformatics investigations revealed that Sertad2 and PPARγ are possible targets of miR-128-3p (44). Chen et al. showed that the expression of miR-128-3p was significantly decreased during the development of 3T3-L1 preadipocyte (mouse embryo source) (44). The high expression of miR-128-3p decreased the expression of adipogenesis biomarkers as well as the formation of lipid droplets and triglyceride contents, indicating the relevance of miR-128-3p in adipogenesis (44). Furthermore, in 3T3-L1 preadipocytes, miR-128-3p appears to suppresses cell proliferation potentially. As a potent inhibitor of adipogenesis, miR-128-3p may selectively target PPARγ, reducing the growth of 3T3-L1 preadipocytes, and miR-128-3p may interact with Sertad2 to induce breakdown triglycerides and lipolysis (44). Overall, these results provided new knowledge about miRNA-mediated proliferation, lipid metabolism, and differentiation processes.

It has been suggested that the expression of miR-128 is involved in the inflammatory response in the periodontal tissues of periodontitis patients. It is also shown that the upregulation of miR-128 can reduce the production of tumor necrosis factor (TNF) and prevent the phosphorylation of p38. It also alleviates the development of macrophages with an inflammatory phenotype (45). Furthermore, increasing evidence suggests that miR-128 might promote neuroinflammation by downregulating PPAR-γ to enhance amyloid beta-induced decreased neuronal survival in Alzheimer’s disease (AD) cells and animal models of AD. Also, it has been reported that miR-128 significantly impacts AD pathogenicity (46–48). In one study, Zhang and colleagues discovered that miR-128 was significantly increased in blood samples from patients with AD compared to healthy controls (49). In summary, they found that miR-128 may be used as a potential biomarker in the serum of patients with AD. Also, this miRNA can be used as a new therapeutic target of neuroinflammation.

## 3 miR-128 biogenesis and targets

miR-128 is produced in two major transcripts through two separate genes, miR-128-1 and miR-128-2, both of which translate into an equivalent mature miRNA sequence. These miRNAs are found in the intronic region of two distinct genes on separate chromosomes. According to studies, miR-128 exhibits organ- and development-specific expression profiles. miR-128 has been identified in the thymus, brain, and skeletal muscle and is found at high concentrations during neural development (21).

According to a study by Mi and colleagues, intronic miR-128-2, which is located in an intron of cAMP-regulated phosphoprotein 21 (ARPP21), was significantly upregulated in all subjects but not in acute myeloid leukemia (AML) (50). Surprisingly, higher miR-128-2 expression was also not associated with increased gene copy number or ARPP21 promoter hypomethylation. Different processes may explain the contradictory expression of miRNA and its host gene. Abnormal expression of miR-128 is common in human cancers. However, depending on the type of cancer, it is highly effective in acting as a tumor suppressor miRNA or oncomiR (51, 52). In addition, it has been shown that miR-128 regulates Long-Interspaced Element-1 (LINE-1 or L1) by connecting directly with open reading frame (ORF) 2 L1 RNA, which encodes L1 RT. (53). Suppression of the L1 element is a driving mutation throughout tumor formation and development (54, 55). In a study, Guzman and colleagues discovered miR-128 as a modulator of telomerase activity in HeLa cells in an anti-miR screen, indicating that miR-128 suppresses endogenous production of telomerase activity (52). Furthermore, they discovered that upregulation of miR-128 decreased telomerase reverse transcriptase (TERT) levels (both mRNA and protein concentrations), whereas reduction of miR-128 increased TERT (both mRNA and protein concentrations) in many cell lines compared to the control group. Finally, they show that miR-128 modulates telomerase activity and affects two sites in the coding region of TERT mRNA. The results indicate that the tumor suppressor miR-128 influences cancer cell oncogenicity through modulating telomerase.

## 4 miR-128 and cancer

Current research has linked the aberrant expression of specific miRNA genes to aggressive disease manifestations, such as malignancy (56). miRNAs act as tumor suppressors or oncogenes based on their modulatory effect on the expression of their target genes (57). Decreased expression of miR-128 has been shown in a variety of cancers such as MLL-AF4 ALL, lung cancer, glioblastoma, and neuroblastoma (**Table 1**) (18, 24b;^92^–^94^). Consequently, the functions and processes of miR-128 in various malignancies will be discussed in this section.

**Table 1:** The role of miR-128 in various types of cancer.

Type of cancer	Sample type	Mechanisms	Findings	Ref.
**BC**	*In vitro*	CDK4/CDK6/Cyclin D1 and CDK2/Cyclin E1 *via* targeting LIMK1	miR-128-3p modulated the LIMK1/CFL1 signaling pathway, which affected BC molecular growth.	(58)
**BC**	*In vitro*	TGF-β signaling	MiR-128a regulated TGF-β activity and lifespan of letrozole-resistant cell culture.	(59)
**BC**	*In vitro*	TGF-β signaling	TGF-β1 modulated MET- and HGF-induced cell motility in BC cell lines and TNBC tissue through positive modulation of C-ets-1 and negative regulatory control of miR-128-3p transcription.	(60)
**BC**	*In vitro* and *in vivo*	Downregulation of HIC1	miR-128 suppressed HIC1 expression and accelerated BC progression.	(61)
**BC**	*In vitro* and *in vivo*	Upregulation of FOXQ1	PVT1 lncRNA induces EMT by upregulating FOXQ1 through miR-128-3p. Furthermore, PVT1 interacts with UPF1 protein and induces EMT, proliferation, and metastasis in BC cells.	(62)
**BC**	*In vitro* and *in vivo*	AurkA-Wnt3a signaling	AurkA repressed the expression of miR-128, an inhibitor of wnt3a mRNA stabilization.	(63)
**BC**	*In vitro* and *in vivo*	Suppression of Wnt signaling the pathway by down-regulating NEK2	miR-128-3p suppressed the stem cell properties of BCSCs by inhibiting the Wnt signaling pathway by downregulating NEK2 expression, providing a new target for BC therapy.	(64b)
**BC**	*In vitro*	Targeting of metadherin	miR-128 was shown to be pathologically upregulated in BC samples and cell lines and was found to be inversely associated with histological grade and cellular tumor progression.	(65)
**BC**	*In vitro* and *in vivo*	Inhibition of the insulin receptor and insulin receptor substrate 1	miR-128 inhibited mitochondrial respiration, glucose metabolism, and growth of TNBC cells. These results were for insulin receptors targeted by miR-128 and suppression of insulin receptor precursor 1.	(66)
**BC**	*In vitro* and clinical	Bmi-1 and ABCC5 overexpression	Stem cell-like specificity of BT-ICs is reduced in miR-128 produced by upregulation of Bmi-1 and ABCC5, leading to chemotherapy drug tolerance in BC.	(23)
**Lung**	*In vitro*	–	miR-128-3p in whole blood served as a novel marker for lung cancer diagnosis.	(67)
**Lung**	*In vitro* and *in vivo*	c-met/PI3K/AKT pathway	miR-128/c-met axis increased the sensitivity of lung cancer stem cells to gefitinib through inhibition of the PI3K/AKT pathway.	(68)
**Lung**	*In vitro* and *in vivo*	Wnt/β-catenin and TGF-β signaling	miR-128-3p was identified as a potential candidate in NSCLC for metastasis and chemoresistance.	(69a)
**Lung**	*In vitro* and *in vivo*	Targeting of VEGF-C and block ERK, AKT, and p38 signaling	miR-128 was fully functional in NSCLC tumorigenesis, partly by modulating lymphangiogenesis and revascularization by targeting VEGF-C and could simultaneously inhibit ERK, AKT, and p38 signaling pathways.	(25)
**Lung**	*In vitro*	EGFR expression	miR-128-b modulated the EGFR transcription in NSCLC cells.	(70)
**Lung**	*In vitro*	E2F5	The involvement of miR-128-2 as a critical role in regulating NSCLC chemoresistance was discovered.	(71)
**Lung**	*In vitro*	SPTAN1	miR-128-3p induced cell cycle arrest and genomic instability in mitomycin C-treated lung cancer cells through inhibition of SPTAN1, and these findings may be used in adjuvant lung cancer therapy.	(48)
**Lung**	*In silico* and *in vitro*	SNAIL and ZEB1	Downregulation of miRNAs through miR-128-3p associated with abnormal expression of SNAIL and ZEB1 promotes the EMT program. This work elucidates the role of miR-128-3p as a major tumorigenic effector of lung cancer cells.	(72)
**Lung**	*In vitro*	MIAT/miR-128-3p/PELI3	This study highlighted the molecular role of the MIAT/miR-128-3p/PELI3-dependent pathway in NSCLC.	(73)
**Glioma**	*In vitro* and *in vivo*	H3K27me 3 and Akt phosphorylation and up-regulation of p21 CIP1 levels, and Bmi-1 down-regulation	miR-128 specifically inhibited glioma self-renewal, which was associated with decreased Bmi-1 expression.	(74)
**Glioma**	*In vitro* and *in vivo*	Targeting of p70S6K1	Recent research has discovered the function and process of miR-128 in controlling glioma neovascularization through the miR-128/p70S6K1 pathway, and miR-128 may be a potential therapeutic target in glioblastoma.	(75)
**Glioma**	*In vitro*	Targeting of NEK2	Downregulation of miR-128 expression by targeting NEK2 reduced glioma cell death.	(76)
**Glioma**	*In vitro* and *in vivo*	LncRNA PVT1 *via* miR-128-3p/GREM1Axis	LncRNA PVT1 upregulates miR-128-3p-regulated downstream signal transduction molecules GREM1 and BMP and promotes malignancy and glioma development.	(77)
**Glioma**	*In vitro*	Upregulation of RhoE	In U251 cells, aberrantly produced miR-128 modulates apoptosis and proliferation by targeting RhoE.	(78)
**Glioma**	*In vitro* and *in vivo*	LncRNA NEAT1 *via* miR-128-3p/ITGA5 Axis	The NEAT1/miR-128-3p/ITGA5 axis is essential in the genesis and development of glioma and may be a viable innovative technique for glioma treatment.	(79)
**Glioma**	*In vitro*	Targeting of PDK1	miR-128-3p/PDK1 axis was important in tumor cell metabolism and proliferation in glioma cells.	(80)
**GBM**	*In vitro* and *in vivo*	Targeting c-Met and EMT	miR-128-3p increased GBM sensitivity to temozolomide by modulating c-Met/EMT.	(81)
**GBM**	*In vitro* and *in silico*	miR-128-3p/ RUNX1/MRP1 axis	RUNX1 conferred temozolomide tolerance in GBM by increasing the expression of MRP1, which is inversely controlled through miR-128-3p.	(82)
**GBM**	*In vitro*	Rap1B	Upregulation of miR-128 attenuated GBM tumor progression by targeting the cytoskeleton and related Rap1B-mediated molecular changes.	(83)
**Neuroblastoma**	*In vitro*	Reelin and DCX	This research concluded that miR-128 functions in the molecular mechanisms that regulate the development and aggressiveness of neuroblastoma.	(24a)
**Neuroblastoma**	*In vitro*	NTRK3 and BCL2	miR-128 modulation of NTRK3 was isoform-specific, suggesting that neurotrophic-mediated activities are closely related to miRNA-dependent processes.	(84)
**Neuroblastoma**	*In vitro* and *in vivo*	SNHG16/ miR-128-3p/HOXA7	SNHG16 transformation reversed the effect of miR-128-3p on neuroblastoma tumorigenesis, motility, invasion, and death.	(85)
**Thyroid**	*In vitro* and *in vivo*	SPHK1	miR-128 may be a tumor suppressor miRNA involved in developing thyroid cancer.	(86)
**Thyroid**	*In vitro*	HCP5	Knockdown of HCP5 *via* miR-128-3p sponge exerted an anticancer effect in ATC, suggesting a possible therapeutic strategy for ATC.	(87)
**OS**	*In vitro*	LncRNA/miR-128-3p/VEGFC axis	The MIAT/miR-128-3p/VEGFC pathway contributed to the development of osteosarcoma and may even be used as a potential therapeutic target for OS.	(88)
**Leukemia**	*In vitro*	–	miR-128 can reliably differentiate ALL from AML, suggesting that epigenetic control may play a key role in maintaining miRNA expression in ALL.	(50)
**Leukemia**	*In vitro*	–	Decreased expression of miR-128b is associated with glucocorticoid tolerance, and restoration of their levels may be an effective treatment in MLL-AF4ALL.	(19)
**Leukemia**	*In vitro* and *in vivo*	HCP5/miR-128-3p/PLAGL2 *via* Wnt/β-catenin/cyclin D1 signaling	HCP5/miR-128-3p/PLAGL2 signaling was associated with an increased risk of multiple myeloma through altered Wnt/-catenin/cyclin D1 signaling.	(37, 89)
**Melanoma**	*In vitro* and *in vivo*	p38 MAPK signaling	miR-128 enhanced DC anticancer immunity by targeting p38 MAPK signal transduction.	(22)
**Laryngeal**	*In vitro* and *in vivo*	–	miR-128a inhibited laryngeal cancer cell proliferation and induced apoptosis.	(90)
**Head and neck carcinoma**	*In vitro* and *in vivo*	–	miR-128 inhibited the growth of HNSCC by directly influencing the expression of potential targets and acting as a tumor suppressor.	(91)

miR-128, miRNA-128; TGF-β, transforming growth factor beta (TGF-β), LIMK1, LIM domain kinase 1; CFL1, Cofilin 1; BC, breast cancer; HIC1, hypermethylated in cancer 1; HGF, hepatocyte growth factor; FOXQ1, Forkhead Box Q1; EMT, epithelial-mesenchymal transition; PVT1, plasmacytoma variant translocation 1; lncRNAs, Long noncoding RNAs; BCSCs, BC stem cells; Bmi-1, B lymphoma mouse Moloney leukemia virus insertion region 1; ABCC5, ATP Binding Cassette Subfamily C Member 5; TNBC, Triple-Negative Breast Cancer; PI3k, Phosphatidylinositol 3-Kinase; VEGF, Vascular endothelial growth factor; NSCLC, Non-small-cell lung carcinoma; ZEB1, Zinc Finger E-Box Binding Homeobox 1; SPTAN1, spectrin-1; MIAT, myocardial infarction-associated transcript; NEK2, NIMA Related Kinase 2; GBM, Glioblastoma; RUNX1, Runt-related transcription factor 1; OS, Osteosarcoma; ALL, Acute lymphocytic leukemia; AML, Acute myeloid leukemia; DC, dendritic cell; HCP5, HLA Complex P5; MAPK, mitogen-activated protein kinase; PLAGL2, Pleomorphic adenoma gene like-2; HNSCC, head and neck squamous cell carcinoma.

### 4.1 Breast cancer

Cancer metastasis is responsible for a significant portion of cancer deaths, and treatments are inadequate, and breast cancer (BC) is no exception (95). About 15% of patients with BC have distant metastases, usually to the brain, liver, lungs, and bones, and 90% of these people will die of metastasis (96, 97). Nevertheless, the processes behind metastatic dissemination remain unknown and represent a significant obstacle to the treatment of BC. In women, BC is the most common type of cancer worldwide (95). BC currently affects approximately 1.7 million individuals worldwide, significantly affecting public health (95). In BC, it was discovered that the expression of miRNAs was altered in different mechanisms of tumorigenesis by modulating different components in distinct signal transduction (98). Several miRNAs, including components of the miR-200 family, are associated with critical pathways of cancer development, including EMT and metastasis in BC (99). It has been found that miR-128-3p can reduce proliferation, differentiation, and motility in BC cells (58). Meanwhile, upregulation of miR-128-3p may affect cell cycle stages by suppressing the production of CDK2/Cyclin E1 and CDK4/6/Cyclin D1. Furthermore, it was found that miR-128-3p may suppress the LIM domain kinase 1 (LIMK1) signaling pathway in BC by targeting the LIM domain kinase 1 (LIMK1) gene (58). These findings point to a novel regulatory mechanism of miR-128-3p-LIMK1/CFL1 in BC, which may lead to new therapeutic approaches for BC.

Metastasis-related miRNAs acting as favorable or unfavorable regulators are known as “metastamirs” (100). Cao and colleagues showed that miR-128, a metastamir, significantly decreased expression levels in human BC samples, which was inversely related to tumor grade, with decreased expression levels higher in grade III (95). A significant correlation was reported in highly aggressive BC cell lines that showed relatively low expression levels of miR-128 (95). Wound healing assays, traditional or dynamic transwell invasion and migration assays, and other functional investigations revealed that aberrant expression of miR-128 in MDA-MB-231 cells [a triple-negative breast cancer (TNBC) cell line] significantly decreased cell migration and invasive potential (95). Moreover, Metadherin (MTDH), an oncogene that regulates bioactivities including apoptosis, longevity, cell metabolism, and revascularization, was discovered to be a specific target gene of miR-128 and is implicated in the miR-128-mediated reduction of initiation and progression in BC cells (95). These data show that miR-128 plays an essential part in BC metastasis and might be a prospective candidate for anti-metastasis treatment.

TNBC is a variant of BC responsible for about 15% of all cases of BC (101). Xiao and colleagues discovered that reduced expression of miR-128 was associated with a shorter lifespan and disease-free survival in TNBC patients but not with a significantly shorter lifespan (66). The finding that limited lifespan in TNBC is associated with reduced miR-128 expression suggests that the involvement of miR-128 in TNBC is consistent with a tumor suppressor, and miR-128 suppressor targets may be oncogenic (66). The involvement of miR-128 in TNBC cells decreased glucose metabolism and inhibited cell growth. It is important to note that glucose metabolism in tumor cells significantly affects cell proliferation. It is unclear whether the reported suppressed cell proliferation is caused by low glucose metabolism or a logical consequence of miR-128 upregulation. However, the finding that miR-128 inhibits the growth of TNBC cells is consistent with the decreased expression of miR-128 in TNBC tissues (66). According to the results of this research, miR-128 may be a suitable biomarker and treatment option in people with TNBC.

**Figure 2:**
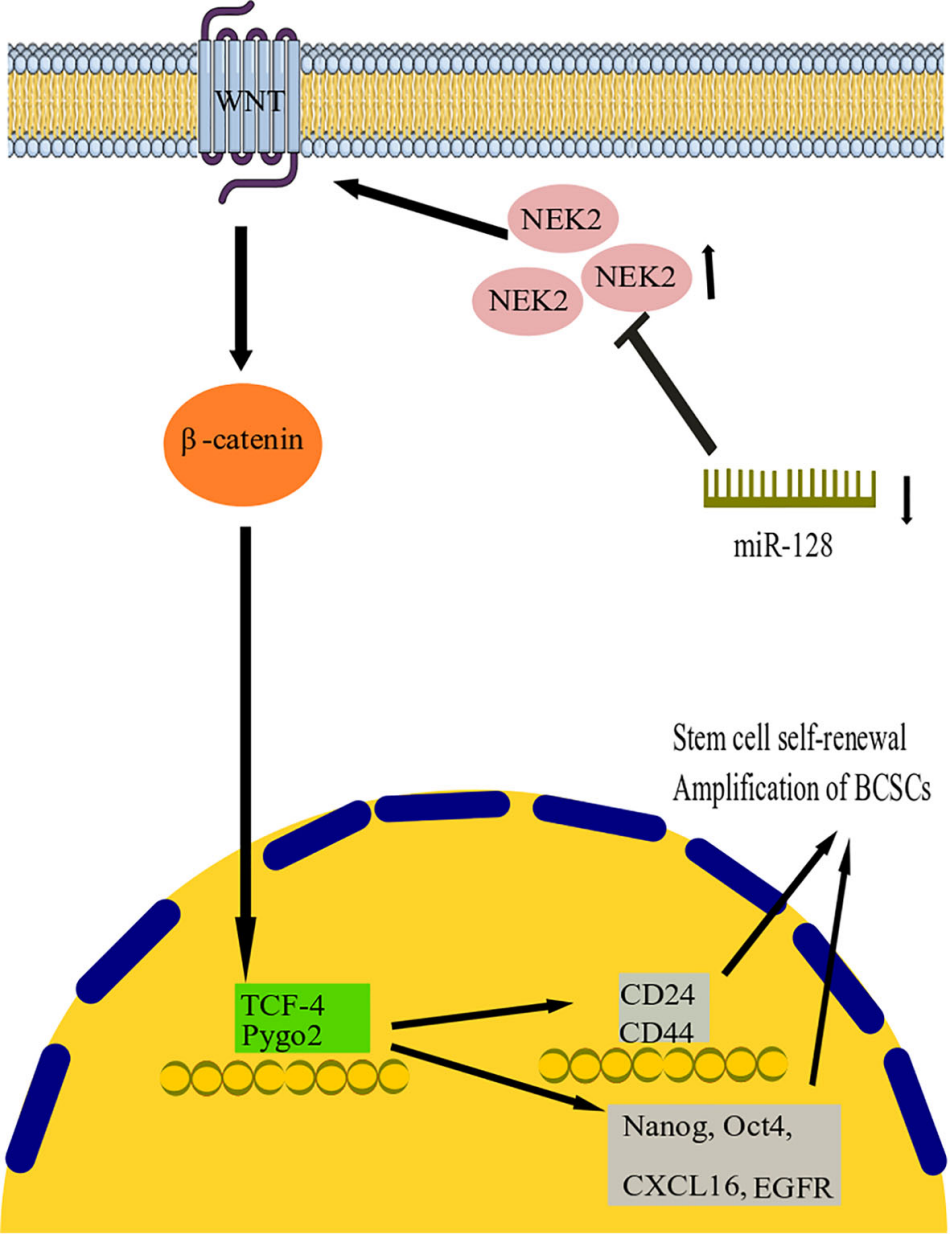
miR-128 action mechanism in breast cancer. It has been found that by reducing NEK2 expression, miR-128 can reduce stem cell properties such as division, motility, invasion, and self-renewal in BC stem cells (BCSCs). The study shows that overexpression of miR-128 reduced BCSC development, motility, and invasion by downregulating the Wnt signaling pathway by downregulating NEK2 expression. miR-128, microRNA-128; BCSCs, Breast cancer stem cells; NEK2, NIMA-related kinase 2; TCF4, transcription factor 4.

Wnt signaling pathways are related to stem cell growth, self-renewal, and migration. They are commonly considered a target for treating several tumor types (102). Activation of the Wnt signaling pathway may promote the cancer growth of BC cells (103). NIMA-related kinase 2 (NEK2) is a type of mitotic kinase involved in tumorigenesis and cancer progression (64). Overexpression of NEK2 in various types of cancer suggests that it could be a potential anticancer drug target (104). Furthermore, a previous study showed that BC’s NEK2 expression is often overexpressed (105). However, both miR-128-3p and NEK2 have been investigated in BC progression, and their exact function in this disease is currently unknown (106, 107). Consequently, Chen and colleagues studied the association between miR-128-3p and NEK2 and its contribution to BC progression in research (64). They discovered that by reducing NEK2 expression, miR-128-3 might reduce stem cell properties such as division, motility, invasion, and self-renewal in BC stem cells (BCSCs) (**Figure 2**). Recent research showed that overexpression of miR-128-3p reduced BCSC development, motility, and invasion by downregulating the Wnt signaling pathway through downregulating NEK2 expression (64). This work established the promising clinical function of miR-128-3p and NEK2 in treating BC through modulating the Wnt signaling pathway. However, the research is still in its early stages, and more research on the molecular mechanism is needed. With increasing technology and a deeper understanding of cancer pathogenesis, more tumor-associated genes have been discovered, potentially providing new targets for therapeutic agents (108). These are hypermethylated in cancer 1 (HIC1), which encodes a transcriptional repressor with multiple partners and targets and is involved in various cancer functions, including cell longevity, proliferation, and migration (109, 110). HIC1 is consistently suppressed in human malignancies such as BC, PC, CC, lung, and liver cancer, thought to be due to promoter hypermethylation (110–113). In their investigation, Li and colleagues discovered that miR-128 was highly elevated in BC tissues (61). Their results suggest that miR-128 could function as an oncomiR in the etiology of BC. Mechanistic studies revealed that miR-128 might effectively bind the 3 untranslated regions (3’-UTR) of HIC1, downregulate its expression, enhance invasion and metastasis, and block apoptosis in BC cells (61). More importantly, restoring HIC1 expression with enhancer plasmids restored miR-128-induced cellular phenotypes, implying that HIC1 targeting is a major determinant through which miR-128 exerts its oncogenic effect. Furthermore, miR-128 control of HIC1 might elucidate, at least in some part, how miR-128 overexpression increases cell invasion and metastasis while inhibiting apoptosis in BC (61a). In conclusion, our findings point to a novel axis consisting of HIC1 and miR-128 that may lead to BC development and provide new possible directions for future BC therapy.

### 4.2 Lung cancer

Lung cancer is a complex disease classified as non-small cell lung cancer (NSCLC) or small cell lung cancer (SCLC) based on pathophysiological features, and NSCLC accounts for 80-85% of all lung malignancies (67). As previously mentioned, dysregulation of miR-128 expression has been documented in various types of human cancers, suggesting that it plays an important role in carcinogenesis, and its role has been explored from tumor suppressor to tissue protumor. Accordingly, it has been found that mutant p53 induces miR-128-3p and its host gene ARPP-21, leading to p53 mutation-mediated chemoresistance in NSCLC and an oncogenic function for miR-128-3p shows in lung cancer (71). Donzelli and colleagues (71) observed that expression of miR-128-2 in lung cancer cells reduces cell apoptosis and induces tolerance to 5-fluorouracil, cisplatin, and doxorubicin treatments. miR-128-2 post-transcriptionally targets E2F5, leading to the loss of its inhibitory function on p21waf1 transcription (71). p21waf1 protein is found in the cytoplasm and has an anti-apoptotic effect by preventing the degradation of procaspase 3 (71). The above findings imply that miR-128-2 regulation promotes mutant p53His175 gain-of-function activities by increasing the multidrug resistance of lung cancer cells.

In a study, Frixa and colleagues showed that miR-128-3p has a direct and suppressive binding effect on Drosha and Dicer 3’-UTRs, leading to an overall downregulation of miRNA expression in NSCLC cells (72). The overexpression of miR-128-3p lowers the abundance of miRNAs targeting important EMT elements, which eventually enhances the aggressive capabilities of the cells transfected (72). In addition, reintroducing Drosha to such a cellular environment led to the restoration of migratory phenotypes, suggesting that Drosha plays an essential function in regulating lung cancer cell motility (72b). These results suggest that miR-128-3p-mediated deletion of Drosha and Dicer transcription may lead to the growth and metastasis of lung cancer cells by indirectly affecting the levels of several functional miRNAs.

**Figure 3:**
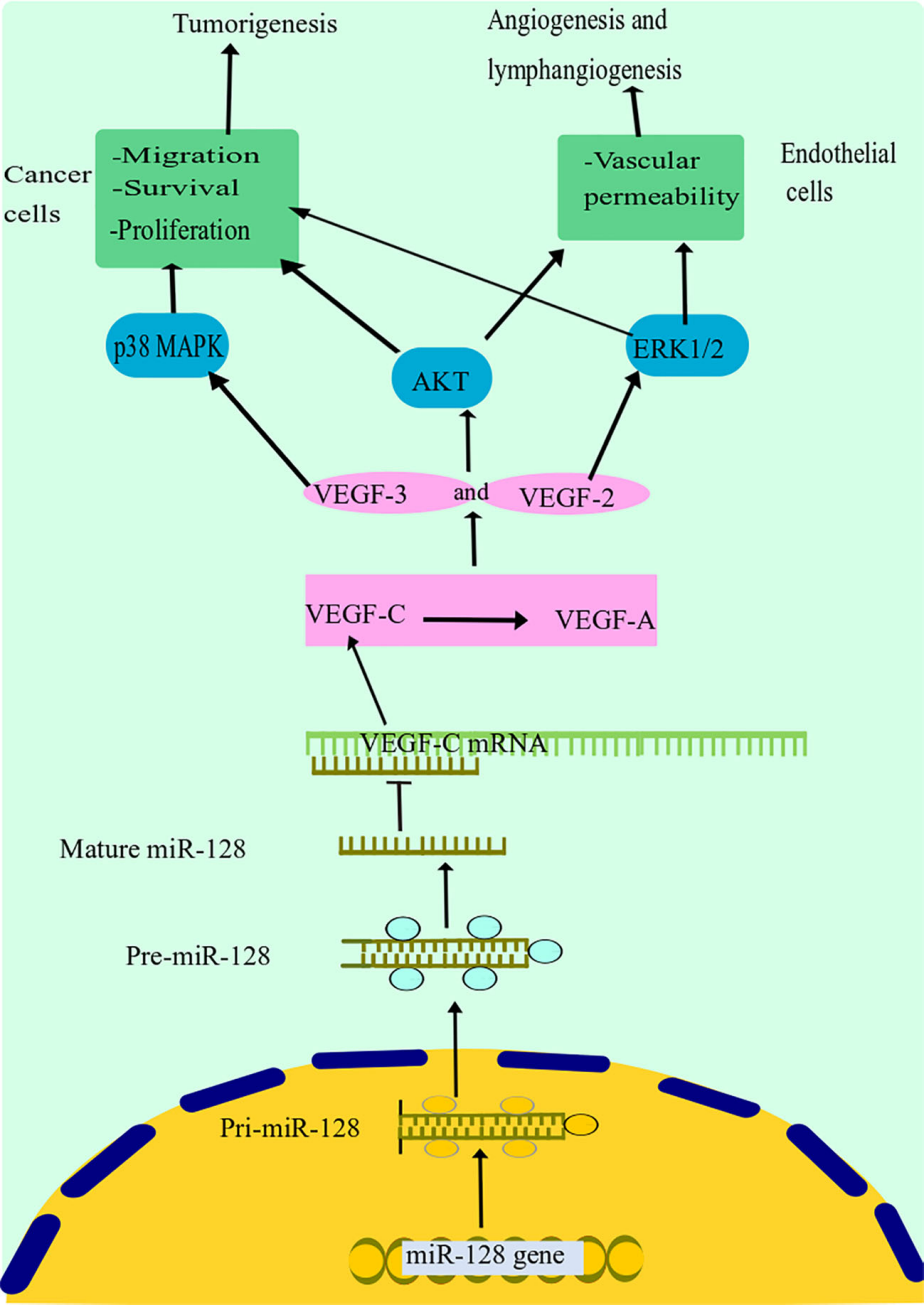
miR-128 suppression mechanism in lung cancer. miR-128 dysregulation dramatically inhibited the expression of VEGF-C. Moreover, upregulation of miR-128 in NSCLC resulted in decreased expression of VEGF-A, VEGF receptor (VEGFR)-2, and VEGFR-3, all of which are important factors in cancer lymphangiogenesis and tumorigenesis, as well as decreased phosphorylation of the phosphatidylinositol 3-kinase and extracellular signal-regulated kinase (ERK). Additionally, it has been discovered that miR-128 may have a function in NSCLC carcinogenesis, partly through the modulation of lymphangiogenesis and angiogenesis by targeting VEGF-C and concurrently blocking ERK, Akt, and p38 signal transduction pathways. miR-128, microRNA-128; VEGF, vascular endothelial growth factor; NSCLC, non-small cell lung cancer; Akt, protein kinase B.

Hu and colleagues discovered that miR-128 expression was highly diminished in tissues and cells of NSCLC and was strongly associated with NSCLC differentiating lymph node metastasis and cancer stage (25). The overexpression of miR-128 remarkably decreased *in vitro* growth, invasion, migration, and colony formation of NSCLC cells and triggered G1 arrest and death. Remarkably, miR-128 dysregulation dramatically inhibited the expression of vascular endothelial growth factor (VEGF)-C as well as reducing the activity of a luciferase reporter, including the untranslated domain of VEGF-C (**Figure 3**) (25). Moreover, upregulation of miR-128 in NSCLC and human umbilical vein endothelial cells (HUVECs) resulted in decreased expression of VEGF-A, VEGF receptor (VEGFR)-2, and VEGFR-3, all of which are important factors in cancer lymphangiogenesis and tumorigenesis, as well as decreased phosphorylation of the phosphatidylinositol 3-kinase and extracellular signal-regulated kin (ERK) (25). Additionally, they discovered that restoring miR-128 *in vivo* significantly reduced the invasion and metastasis of A549 cells in nude mice and decreased both lymphangiogenesis and revascularization in tumor xenografts (25a). The above data support the hypothesis that miR-128 may have a function in NSCLC carcinogenesis, partly through the modulation of lymphangiogenesis and angiogenesis by targeting VEGF-C and concurrently blocking ERK, protein kinase B (PKB), also known as Akt, and p38 signal transduction pathways. Targeted therapies to reestablish miR-128 in NSCLC may be effective in inhibiting tumor development.

Notably, miR-128-3p antagonism profoundly affects metastasis and chemoresistance in aggressive phenotype NSCLC cells, which can be entirely reversed by restoring Wnt/β-catenin and TGFβ-activities, revealing that miR-128-3p might be a candidate for both tumor growth and chemoresistance in NSCLC (69b). In the current work, Cai et al. induced a model of NSCLC xenografts (chemoresistance-associated cancer progression). They showed that in several NSCLC cell lines, cancer stem cell (CSC) programming and EMT are driven by miR-128, which indicates tumorigenesis, chemoresistance, and disease progression through simultaneous activation of β-catenin and TGF-β signaling (69b). Remarkably, they discovered that reactivation of TGF-β and Wnt/β-catenin signaling pathways restored the antagonistic action of miR-128-3p on chemoresistance and proliferation in highly aggressive NSCLC cells (69b). As a result of these discoveries, it is possible to block both pathways simultaneously by targeting a single molecule to improve chemoresistance and metastasis in NSCLC.

According to the literature, miR-128-3p expression is decreased in lung cancer tissue compared to normal tissue (114). Nevertheless, the therapeutic significance of this miRNA in the early detection of lung cancer is unknown. Pan and colleagues discovered that the expression levels of miR-33a-5p and miR-128-3p were decreased in lung cancer cell lines and tissues (115). They identified that the expression levels of miR-33a-5p and miR-128-3p in lung cancer tissues were substantially linked with the tumor, node, and metastasis (TNM) grades. Significantly, the levels of miR-128-3p and miR-33a-5p in the blood of patients with lung cancer or initial lung cancer subjects (TNM grade I-II) were lower than in normal participants (115). Receiver operating characteristic curve (ROC) analysis revealed that miR-33a-5p and miR-128-3p, alone or in combination, had higher AUC scores and enhanced sensitivity/specificity than conventional biomarkers in their study (115). Remarkably, although under severe conditions, miR-128-3p and miR-33a-5p were very stable in blood. These findings suggest that miR-33a-5p/miR-128-3p in the blood may be used as new markers for lung cancer diagnosis. Mitomycin C (MMC), a potent DNA cross-linker, acts against NSCLC, a process that requires interstrand DNA cross-linking to inhibit the replication and proliferation of malignant cells (116). Although II Sp is involved in the repair of DNA interstrand cross-links (DNA ICLs) as a structural protein, the interaction between miRNAs and SPTAN1 in DNA repair and the potential role of MMC in suppressing tumor cells are unknown. Zhang et al. revealed a unique function for miR-128-3p in MMC-exposed lung cancer cells, where chromosomal viability and cell cycle progression are regulated through SPTAN1 (116).

In summary, these findings add to our understanding of the dynamics of miR-128-3p in lung cancer sensitivity to chemotherapy. The miR-128-3p-SPTAN1 axis opens up a new window into the chemosensitivity process, and miR-128-3p may be a possible molecular candidate to improve the lung chemotherapy process. Finally, while these studies have shown that miRNA deregulation is responsible for chemoresistance, the function of miRNAs in controlling CSC chemoresistance is unclear. In this regard, Jiang et al. discovered that in lung cancer cells, the expression of miR-128 is reduced, which is associated with gefitinib tolerance in these cells (68). Increased expression of miR-128 was later reported to increase PC9-CSC sensitivity to gefitinib, thus limiting the efficacy of gefitinib in enhancing the CSCs population *in vitro* and *in vivo* (68). Furthermore, they discovered that gefitinib did not inhibit the Phosphoinositide 3-kinase (PI3K)/AKT pathway in PC9-CSCs.These results suggest that the miR-128/c-met axis improves gefitinib susceptibility in lung cancer stem cells by inhibiting the PI3K/AKT axis.

### 4.3 Thyroid cancer

Thyroid cancer is a frequent endocrine cancer that has been rising globally over the last several decades (117). Follicular thyroid carcinoma (FTC) and papillary thyroid carcinoma (PTC) (well-differentiated), and also anaplastic thyroid carcinoma and imperfectly differentiated, are the histotypes of thyroid cancer (118, 119). Despite substantial research on the role of genetic defects and environmental variables in thyroid tumorigenesis, the specific molecular pathways behind the development of thyroid malignancy remain unclear. Cao and colleagues discovered that miR-128 expression was significantly decreased in tissue and several human PTC and FTC cell lines (86). Restoration of miR-128 activity in PTC and FTC cells significantly reduced cell survival, motility, and invasion. In addition, upregulation of miR-128 induced cell cycle arrest in G0/G1 phase and apoptosis. Sphingosine kinase 1 (SPHK1) was also a primary target of miR-128 (86). SPHK1 regulates cellular activities and tumorigenesis, including reproduction, apoptosis, and proliferation (120). Cao et al. found an inverse correlation between SPHK1 and miR-128 expression in FTC and PTC samples, and luciferase reporter assays and RT-qPCR analyzes showed that miR-128 downregulates SPHK1 expression by targeting its 3’UTR (86). In addition, they discovered that up-regulated miR-128 suppressed cancer growth *in vivo* and was found to be a tumor suppressor in thyroid cancer. Also, recent research shows that 5-aza-20-deoxycytidine significantly increases the expression of miR-128 in thyroid cancer cell lines, suggesting that miR-128 plays a vital role in regulating thyroid cancer proliferation (86).

Furthermore, the researchers discovered that upregulation of miR-128 significantly increased apoptosis in thyroid cancer cell lines, primarily through overexpression of caspase-3 and polyadenosine diphosphate-ribose polymerase (PARP) was confirmed. Upregulation of SPHK1 inhibits miR-128-induced apoptosis, suggesting that SPHK1 may contribute to miR-128-regulated apoptosis (86). These findings showed that in PTC and FTC tissue or cells, the expression of miR-128 was decreased, and SPHK1 was increased. Based on functional experiments, apoptosis and cell cycle arrest in G0/G1 phase were observed after restoring miR-128 expression, which inhibited thyroid cancer progression and also reduced invasion and metastasis. SPHK1 has been discovered to be a primary target of miR-128. According to the findings, miR-128 might be a promising treatment target for thyroid prevention and therapy by decreasing SPHK1.

Anaplastic thyroid carcinoma (ATC) is an uncommon thyroid cancer characterized by rapid growth, extrathyroidal infiltration, and lymph node metastasis to the brain, lungs, and bones (121, 122). ATC has a significant mortality rate, with a median survival time of 5 months and 20% overall survival at one year (123). Human leukocyte antigen (HLA) complex P5 (HCP5) has recently been shown to be a tumor suppressor in the formation of PTC, which accounts for 80–85% of thyroid malignancies (124). On the other hand, the function of HCP5 in ATC is unclear. Chen et al. studied the expression of HCP5 in ATC and determined if HCP5 modulated miR-128-3p in ATC to control ATC cell survival and apoptosis. They found that the expression of miR-128-3p was decreased in the ATC cell line and tissue, and miR-128-3p was the substrate of HCP5 in ATC cells in further experiments (87). Chen and colleagues also found that HCP5 regulates miR-128-3p expression (87). Taken together, the HCP5/miR-128-3p axis plays a critical function in controlling the survival and death of ATC cells, suggesting that HCP5 can be used as a therapeutic approach for ATC operation.

### 4.4 Head and neck cancer

Head and neck cancer (HNC) has become one of the malignancies whose prevalence has increased over the past decade, although survival rates have not increased significantly (125, 126). Squamous cell carcinoma (HNSCC) occurs in the epithelial lining of the nasopharynx, pharynx, larynx, and oral cavity, accounting for more than 90% of HNC (127, 128). Research demonstrates that miR-128 regulates E2 promoter-binding factor a (E2Fa), Bmi-1, and other regulatory regions, including transcriptional WEE1-A (a tyrosine kinase) that phosphorylates CDK1 to promote tumorigenesis (129). Overexpression of miR-128 suppresses HNSCC development by directly modulating its targets, Paip-interacting protein 2 (Paip2), BAG Cochaperone 2 (BAG-2), H3F3B, Bmi-1, and Bcl-2-associated X protein in proliferative and apoptotic processes, indicating that miR-128 acts as a tumor suppressor, *in vitro* and *in vivo*. Hauser et al. examined the function of miR-128 in the control of HNSCC development as well as its potential targets (91). They showed that almost all target mRNAs contain a complementary 3’UTR sequence that may pair with miR-128 to inhibit target mRNA translation and lead to decreased protein levels. Paip2, Bmi-1, and H3F3B proteins are involved in tumor growth, and downregulation of Bmi-1 and H3F3B expression reduces cancer growth and xenograft development in JHU-22 miR-128 cells (91). In addition, Hauser and colleagues discovered that JHU-13 miR-128 inhibited cell growth and confirmed the binding of miR-128 to the 3’UTR of BMI-1 mRNA (91). They also found that the expression levels of cell proliferation regulators were altered with lower protein levels of cyclin D1 and PCNA in JHU-22 miR-128 cells. Current findings suggest that miR-128 is involved in several signaling pathways related to the development and growth of HNSCC. Further research is needed to confirm the expression and activity of miR-128 in HNSCC as well as other pathogenic forms of human cancer. Bmi-1 has been found to contribute to laryngeal squamous cell carcinoma (LSCC) progression and maintain tumorigenic laryngeal growth, suggesting that miR-128 plays a tumor suppressor role in laryngeal cancer (130, 131). In addition, the involvement of miR-128 in this cancer should be confirmed. Wan and colleagues first reported that miR-128a expression is decreased in primary laryngeal cancer and that upregulation of endogenous miR-128a reduces cell growth and increases apoptosis in research (90). In conclusion, according to the latest research, miR-128a is significantly downregulated in LSCC. In addition, they discovered that the upregulation of miR-128a decreased the growth of laryngeal Hep2 cells and accelerated apoptosis (90). Moreover, miR-128a overexpression inhibited cancer progression *in vivo*. As a result, targeting miR-128a might be a unique strategy for treating LSCC.

### 4.5 Osteosarcoma

Osteosarcoma (OS) is the most common type of bone cancer, accounting for 20% of primary bone tumors and the second leading cause of tumor-related deaths among young adults (132, 133). Survival rates remain low, with 80% of surgically treated patients experiencing recurrence or dissemination (134, 135). Consequently, it is important to investigate the molecular processes behind the overall development of survival and develop promising drug options for the treatment of survival rates. Zhang and colleagues investigated the physiological role of its long non-coding RNA (lncRNA) myocardial infarction-associated transcript (MIAT) in survival through miR-128-3p/VEGFC axis control in OS tissues and cell lines (88). It is generally understood that lncRNAs act as ceRNAs and regulate functional gene expression by sponge miRNAs (136). To confirm these findings, Zhang and colleagues used bioinformatics methods to predict MIAT-miRNA and dual luciferase reporter analysis to test the effect of target binding, and they discovered that MIAT is the primary target of miR-128-3p (88). Their findings showed that miR-128-3p transcription was significantly decreased in cell lines and tissues and inversely correlated with MIAT expression in survival rate. The above results suggest that miR-128-3p acts as an inhibitor of survival rate. They used DIANA and TargetScan v 7.2 techniques to predict the target genes of miR-128-3p and discovered that VEGFC is a possible predisposing factor of miR-128-3p.

In addition, recent research has shown that VEGF is a major downstream substrate of miR-128-3p and affects the growth of lymphatic endothelial cells (137). Zhang and colleagues showed that VEGFC expression was found to be negatively related to miR-128-3p expression in survival rate and validated that VEGFC was a primary target of miR-128-3p in MG63 cells, implying that there was a ceRNA circuit in OS between VEGFC, MIAT, and miR-128-3p (88). They showed that suppressing miR-128-3p diminished the effect of MIAT knockdown on VEGFC protein levels and the growth, apoptosis, and metastasis in MG63 cells. According to the findings of this research, the MIAT/miR-128-3p/VEGFC axis may be a unique prospective treatment method to improve the survival rate in OS.

### 4.6 Glioma

About 20,000 new cases of glioma are diagnosed in the United States each year, and even with aggressive surgery, chemotherapy, and radiation therapy, the median survival for the most malignant type (glioblastoma) is approximately 14 months (74, 138). miRNA analysis has revealed different expression profiles in glioblastoma and other human malignancies (139). Several miRNAs have recently been discovered to have a significant function in glioblastoma. miR-7 expression was found to be decreased in glioblastoma, inhibiting the invasion and metastasis of primary glioblastoma lines, whereas miR-26 enhances glioblastoma cancer development *in vitro* and *in vivo* by attempting to target numerous tumor suppressor genes, including RB Transcriptional Co-repressor 1 (RB1) and Phosphatase and tensin homolog (PTEN) (140, 141). The majority of miR-128 research on tumorigenesis has focused on glioblastoma. For example, miR-128 is downregulated in glioblastoma (74, 139). Upregulation of miR-128 reduces cell proliferation by targeting the transcription factor E2F 3a (E2F3a) and Bmi-1 while blocking the Reel and Doublecortin (DCX) promoters reduce neuroblastoma cell migration and metastatic spread (24, 74, 139).

Shi et al. discovered that the miR-128 expression is reduced in glioblastoma and acts as a tumor suppressor by specifically targeting p70S6K1 (75). They determined that miR-128-induced transcription downregulated the expression of VEGF, p70S6K1, and Hypoxia-Inducible-Factor 1α (HIF-1α). Transformation of p70S6K1 restored miR-128-inhibited expression of HIF-1a and VEGF, indicating that p70S6K1 is a target of miR-128 (75). Furthermore, in their research, upregulation of miR-128 inhibited cell proliferation, tumor development, and revascularization *in vivo*. These findings add to our knowledge of the importance and process of miR-128 in glioblastoma pathogenesis and suggest a promising therapeutic approach for the treatment of glioblastoma.

According to a study carried out by Godlewski et al., miR-128 expression is downregulated in glioblastoma, and Bmi-1 is the primary target of miR-128 (74). Upregulation of Bmi-1 was observed in various types of cancer and is a potent stimulator of stem cell regeneration. Also, research on transgenic mice showed that Bmi-1 plays an essential role in the formation of glioblastoma (142–144). These findings show that the effect of miR-128 on glioblastoma cells is consistent with the reduction of Bmi-1 expression, including one with a reduction in self-renewal of glioblastoma stem cells, suggesting that miR-128 may have the potential for clinical translation of glioblastoma stem cells. Shang et al. observed that miR-128 expression was lower in glioblastoma specimens compared to the control group (78). Increased miR-128 expression suppressed U251 cell growth by targeting the RhoE gene at the translational level. The increase in RhoE expression restored the progressive effects of pre-miR-128 on growth and apoptosis in U251 cells (78).

In conclusion, abnormally produced miR-128 modulates apoptosis and reproduction in U251 cells, partially *via* inhibiting RhoE. These data imply that aberrant miR-128 expression is critical for glioblastoma cell death and growth. Additional studies in this area may help to develop new anti-glioma treatment techniques. Ye and colleagues indicated that the miR-128 expression in glioblastoma tissues was much less than in healthy brain tissues (76). miR-128 expression was associated with tumor volume and aggressiveness of glioblastoma in their study but not with the gender or age of glioblastoma patients. However, miR-128 upregulation induced apoptosis in U87 cells and high protein content of degraded Caspase-3, Bcl-2, and Bax (76). The researchers showed that miR-128 was clearly associated with NEK2 using a dual luciferase reporter gene assay, and additional investigations showed that upregulation of NEK2 partially restored the effect of miR-128 on glioblastoma cell death. These findings suggest that upregulated miR-128 prevents apoptosis in glioblastoma cells by targeting NEK2 and contributes to the occurrence and development of glioblastoma.

Qu and colleagues investigated whether and how abnormal expression of miR-128 might alter the metabolic activity of glioblastoma (145). They found that miR-128-3p inhibited lactate synthesis, elevated ROS, and impaired mitochondrial activity in glioblastoma cells by targeting Pyruvate Dehydrogenase Kinase 1 (PDK1). PDK1 is a critical enzyme in converting glycolysis into the tricarboxylic acid cycle by suppressing Pyruvate dehydrogenase and transforming oxidative phosphorylation to the Warburg process, which increases lactate production (145). Suppression of PDK1 expression decreased lactate and ATP levels, increased reactive oxygen species (ROS) formation, mitochondrial dysfunction, decreased cell proliferation, and increased cell death. Their findings indicated that the miR-128-3p/PDK1 axis is important in the metabolism and development of tumor cells (145). These data suggest that pharmacological efforts to control the Warburg effect, including inhibition of PDK1, might be a potential therapy for treating glioblastoma.

Glioblastoma is the most severe type of glioma and the most common invasive and lethal brain tumor in children and adults with a catastrophic prognosis (82, 146). The expression of Runt-related transcription factor 1 (RUNX1) is significantly higher in the mesenchymal subtype of glioblastoma and is strongly linked to the mesenchymal subtype initiated through miRNA-mediated pathways (147). Previous researchers reported that RUNX1 is involved in the aggressive nature of glioblastoma (148). A new study showed that the upregulation of RUNX1 can significantly enhance glioma growth and metastasis (149). It has also been observed that the downregulation of RUNX1 improves temozolomide sensitivity and suppresses glioblastoma growth (150). However, the regulation of RUNX1 expression in glioblastoma remains unknown. A growing body of data indicates that multidrug resistance protein 1 (MRP1), which is upregulated in cancers, modulates cellular chemoresistance, and temozolomide has been identified as a target of MRP1 (151, 152). In conclusion, Zhou and colleagues investigated the underlying mechanisms of temozolomide tolerance and discovered the miR-128-3p/RUNX1 pathway as a novel target for temozolomide tolerance in glioblastoma (82). They confirmed the oncogenic involvement of RUNX1 in glioblastoma cells and discovered miR-128-3p as a potent inhibitor of RUNX1. The researchers also discovered that RUNX1 upregulated MRP1 to induce temozolomide tolerance (82). These findings suggest that miR-128-3p/RUNX1/MRP1 axis modulates temozolomide resistance in glioblastoma cells, and these molecules may be used to regulate temozolomide responsiveness in glioblastoma.

### 4.7 Leukemia and multiple myeloma

ALL and AML are genetically distinct and arise from myeloid blood cells, lymphoid progenitors, or primary stem cells with multilineage potential (153, 154). Because the treatment and prognosis of ALL and AML are significantly different, ALL must be differentiated from AML in the evaluation (155, 156). While ALL and AML may be differentiated using appropriate morphologic, immunohistochemical, and immunological methods, the traditional clinical practice requires competent staff, and currently, no single test is sufficient to diagnose a patient (157). Mi and colleagues performed a genome-wide miRNA expression assessment in a breakthrough study to discover biomarkers for the diagnosis and treatment of ALL and AML and to provide insight into the unique pathways of leukemogenesis between ALL and AML (50). miR-223, miR-128b, miR-128a, and let-7b were the most significant and differentially expressed among the 27 miRNAs expressed between ALL and AML. miR-128a and -128b were significantly more abundant in ALL, while miR-223 and let-7b were significantly more abundant in AML. May and colleagues showed that overexpression of miR-128 in ALL was not associated with duplication of genomic loci compared to AML and normal control samples (50). However, researchers demonstrated that the methylation of CpG islands in the miR-128b promoter was much lower in ALL samples than in AML samples and that there was an inverse correlation between miRNA expression and CpG island methylation.

In conclusion, overexpression of miR-128 in ALLs versus AMLs was related to epigenetic changes, i.e., hypomethylation of CpG islands in the promoter region. Remarkably, Although miR-128 was expressed in almost every AML as well as normal control samples at a significantly reduced level compared to ALL samples, the degrees of miR-128 promoter methylation were almost identical in control subjects and subgroups of AML samples. This suggests that another process influencing expression may warrant further investigation (50). Overall, their work suggests that the expression characteristics of miRNAs such as miR-128 may reliably distinguish ALL from AML and that epigenetic regulation may significantly govern miRNA expression in acute leukemias.

MLL-AF4 ALL, caused by a symmetric translocation between MLL and AF4, accounts for approximately 50% of ALL cases in infants, 2% in children, and 5% to 6% in adults (158). Kotani and colleagues showed that reexpression of miR-128b rendered two MLL-AF4 ALL cell cultures susceptible to death with high and low doses of glucocorticoid and etoposide and serum deprivation (19). They discovered that several miRNAs, such as miR-128b and miR-221, are decreased in MLL-rearranged ALL primary cell samples in comparison to other types of ALL (19). Given their fundamental involvement in virtually all ALL therapies, the mechanism by which glucocorticoids act on their target cells and the molecular pathways that confer glucocorticoid resistance remain largely unknown. In conclusion, miR-128b and miR-221, which restore steroid sensitivity, may provide a clear picture of the process of glucocorticoid activity. Administration of miR-128b and miR-221 to MLL-AF4 ALL leukemia cells *via* a carrier, including a suitable liposome, could complement conventional chemotherapy. Most importantly, these two miRNAs work together to induce chemoresistance. Two chimeric mRNAs, AF4-MLL and MLL-AF4, caused by the disease-causing t(4;11) chromosomal translocation, are two key targets of miR-128b (158). Their findings suggest that miR-128b and miR-221, especially miR-128b, play important roles in lymphoid biosciences. Most importantly, the effects of miR-128b and miR-221 on drug resistance are additive, suggesting that the binding of these miRNAs is a suitable target for treating diseases.

Multiple myeloma is a frequent hematologic malignancy with a significantly higher incidence and mortality rate than non-Hodgkin’s lymphoma (89). Despite recent advances in traditional chemotherapy and stem cell transplantation, the five-year survival rate for people with multiple myeloma remains poor. Consequently, a thorough knowledge of the putative mechanistic interactions involved in multiple myeloma at the genomic/transcriptional levels is essential.

## 5 miR-128 and chemoresistance in cancer

Chemotherapy tolerance remains a key obstacle in successful anti-cancer treatments and causes the recurrence and development of more malignancies (23). Cancer-initiating cells, often called cancer stem cells, are a subpopulation of cancer cells with stem cell-like properties that have recently been identified in a wide range of human cancers, including BC, prostate, brain, liver, pancreas, and blood cancer (159–169). These cells are resistant to several chemotherapy protocols and are considered the leading cause of cancer recurrence after treatment (170–172). However, some biological mechanisms, including overexpression of ATP-binding cassette transporters, and increasing anti-apoptotic and effective DNA damage response, are associated with chemoresistance in cancer cells; None of these processes are stem cell-like properties, and thus their contributions to tumor-initiating cell tolerance therapies remain unresolved (173). In this context, it has been shown that abnormal miRNA expression is engaged in various biological pathways associated with chemotherapy resistance mechanisms. Zhu and colleagues demonstrated that decreasing miR-128 in BC–initiating cells causes upregulation of Bmi-1 and ABCC5, 2 autonomous substrates of miR-128 (23). Overexpression of miR-128 in the setting of doxorubicin lowered cell viability while increasing apoptosis and DNA damage, rendering BC-initiating cells more sensitive to therapy. They additionally discovered that decreased amounts of miR-128 in metastatic BC tissues were associated with poor clinical therapeutic efficacy and survival rates. As a result, decreasing miR-128 in BC-initiating cells leads to chemoresistance by reducing its suppression of Bmi-1 and ABCC5 translation.

Tolerance to hormonal treatment has been identified as a medical barrier in the therapy of hormone-dependent BC (59). Masri and colleagues analyzed the impact of miRNA modulation of aromatase inhibitors on the signaling pathways that lead to the development of BC on aromatase inhibitors (59). Their study of hormone-resistant cell lines found 115 differentially regulated miRNAs, 49 of which were hormone-responsive, including a set of miRNAs that were regulated inversely in aromatase inhibitor-resistant lines compared to long-term estrogen-free lines and tamoxifen-resistant cells. They highlighted the hormone-responsive gene hsa-miR-128a, which was selectively overexpressed in letrozole-resistant cell lines. It has been observed that miR-128a inversely targets TGF RI protein production by binding to the 3’-UTR domain of this gene. After endogenous suppression of miR-128a, letrozole-resistant lines were sensitized to the development of the antagonistic activity of TGF-β. These results suggest that hormone-responsive miR-128a can alter TGF signaling and the survival of letrozole-resistant cell lines.

Zhao and colleagues discovered that the expression of miR-128-3p was significantly decreased in glioblastoma cell lines and tissue. miR-128-3p inhibited glioblastoma proliferation, invasion, and motility and enhanced the therapeutic benefit of temozolomide by suppressing glioblastoma proliferation, invasion, and migratory behaviors and initiating apoptosis (174). miR-128-3p, when combined with temozolomide, reduced tumor size and invasion while increasing glioblastoma sensitivity to temozolomide in tumor-bearing nude mice (174). Recent research elucidates the function of miR-128-3p in enhancing glioblastoma chemosensitivity and the underlying principles. Overall, miR-128-3p may be a suitable tool for identifying drug-resistant therapeutic interventions. Likewise, She et al. found that miR-128 increased temozolomide chemosensitivity *via* Rap1B-mediated cytoskeletal reorganization in glioblastoma (175).

All in all, they showed that the expression of miR-128 was significantly decreased in glioblastoma, suggesting that the decreased expression of miR-128 was involved in the progression of astrocytoma cancer. Upregulation of miR-128 inhibited glioblastoma invasion and dissemination by targeting Rap1B-mediated cytoskeletal remodeling and related substances, including N-cadherin, cell division cycle 42 (Cdc42), and RhoA (175). Studies revealed that miR-128 increased the chemosensitivity of human glioblastoma cells to temozolomide. Consequently, the restoration of miR-128 transcription may be a strategy for treating glioblastoma, and the combination of miR-128 mimics with temozolomide may be a successful therapeutic approach to reduce the development of glioblastoma.

## 6 Role of miR-128 in immune responses and immunotherapy in cancer

Recently, there seems to be much attention on determining the function of miRNAs in modulating anti-tumor immunity and how this may affect the efficacy of various cancer therapies (176, 177). Thus, protective immunity has collateral and anti-oncogenic consequences, and the dynamic interaction between immune and tumor cells in the tumor microenvironment significantly regulates tumor growth. miRNAs regulate many immune-tumor cell junctions and essential immune response processes (178). miRNAs have also been discovered to be tumor suppressors or oncogenes, with the ability to control antitumor immunity or crosstalk between cancer cells and their surrounding immune cells (179). miRNAs can be used as prognostic, diagnostic, and targeted immunotherapeutics based on their specific regulatory role. Consequently, to develop successful and safe miRNA-based anticancer therapeutic options, a thorough analysis of the precise functions of miRNAs in the tumor microenvironment (TME) is essential.

**Figure 4:**
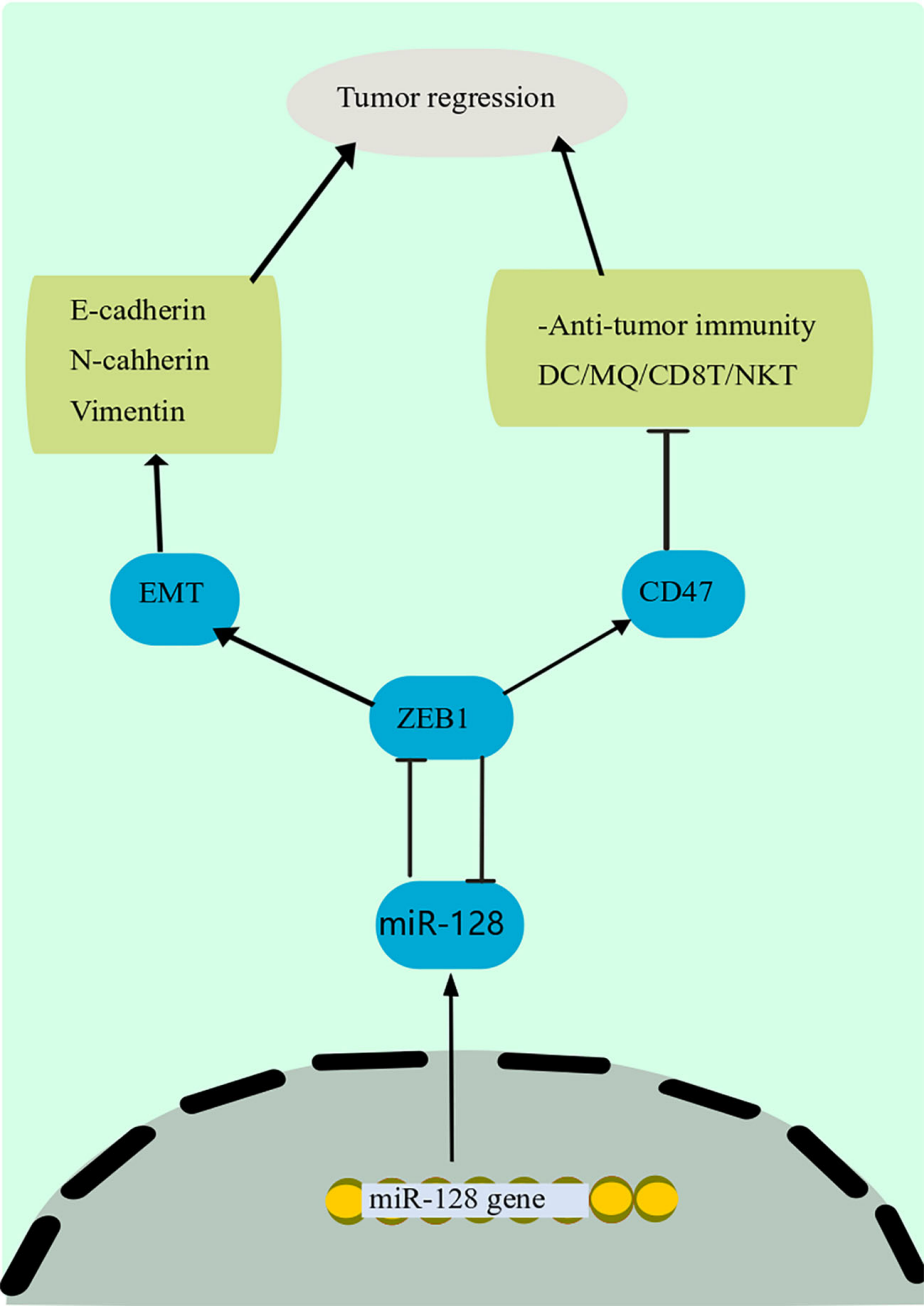
Anti-tumor immunity mechanism of miR-128. miR-128 modulates the invasion of anticancer immune cells in the immunological milieu, comprising DCs, CD8 ^+^ T cells, and NKT cells, *via* the ZEB1/CD47 axis and EMT, eventually suppressing PC development and dissemination. The downstream mechanism of miR-128 in the EMT of cancer cells has been found in a study by targeting genes such as ZEB1. Among these target genes, ZEB1 has been mentioned to regulate the EMT of cancer cells in cervical cancer and esophageal squamous cell carcinoma. DCs, Dendritic cells; miR-128, microRNA-128; NKT, natural killer T; ZEB1, Zinc Finger E-Box Binding Homeobox 1; EMT, epithelial-mesenchymal transition.

A growing body of data suggests that miRNAs influence tumor immune responses, particularly innate and adaptive immune responses (180, 181). In addition, some miRNAs have an important modulatory function in immune cells and cancer cells, supporting tumor immune suppression or creating an immunosuppressive environment (182). Cancer-derived miR-214, for instance, can promote the proliferation of CD4^+^ CD25 high Forkhead Box P3 (FoxP3) ^+^ Regulatory T cells (Tregs) by addressing PTEN and stimulating IL-10 production, resulting in host immune repression and accelerated tumor progression (183). Downregulation of miR-128-3p in gastric cancer has increased cell growth (184). Besides, miR-128 modulates the invasion of anticancer immune cells in the immunological milieu, comprising DCs, CD8 ^+^ T cells, and natural killer T (NKT) cells, *via* the Zinc Finger E-Box Binding Homeobox 1 (ZEB1)/CD47 axis and EMT, eventually suppressing PC development and dissemination (**Figure 4**) (185). The downstream mechanism of miR-128-3p in the EMT of cancer cells has been found in a study by targeting genes such as ZEB1, CDC6, FOXO4, and SCAMP3 (186). Among these target genes, ZEB1 has been mentioned to regulate the EMT of cancer cells in cervical cancer and esophageal squamous cell carcinoma (186). miR-128 has been found to block the p38 MAPK pathway, which inhibits the production and production of IL-6 and IL-10 while increasing the amount of IL-12 in DCs., hence boosting the anti-cancer immunity of DCs and decreasing cancer progression in melanoma (22).

VEGFs are significant revascularization and lymphangiogenesis mediators throughout cancer formation (25). These substances and VEGFRs have been identified as the main therapeutic targets to reduce pathogenic angiogenic and lymphogenic signals (25). Hu et al. showed that the expression of miR-128 was strongly downregulated in NSCLC tissues and tumor cells and strongly correlated with NSCLC differentiation, clinical stage, and distant metastasis (25). In their study, aberrant regulation of miR-128 completely inhibited VEGF-C expression. This action downregulated a luciferase reporter containing the VEGF-C 3’-UTR. Transduction of miR-128 in NSCLC cells and HUVECs resulted in decreased expression of VEGF-A, VEGFR-2, and VEGFR-3, essential requirements to explain lymphangiogenesis and tumor vasculature, and a gradual decrease in the phosphorylation of ERK, AKT, and the p38 axis. The above data imply that miR-128 may play a significant function in NSCLC carcinogenesis, partly through modulating revascularization and lymphangiogenesis *via* addressing VEGF-C and concurrently impeding AKT, p38, and the ERK signal transduction pathways.

Two targeted drugs often used to treat people with advanced NSCLC are monoclonal antibodies and small molecule receptor tyrosine kinase inhibitors (TKIs) (187). Previous research has shown that EGFR-TKIs are a suitable treatment modality that is beneficial for cancers (with mutations in the EGFR gene) (187, 188). Also, increased EGFR gene copy number is associated with a better prognosis for patients receiving TKI therapy. (189). Current findings have also shown that some TKI-responsive individuals have no significant genetic alterations in EGFR (190). Because EGFR-TKIs were effective in 10–30% of people with chemotherapy-resistant NSCLC, identifying novel regulatory molecules may improve targeted lung cancer therapy (191, 192). It has also been reported that decreased heterozygosity in miR-128-b in NSCLC cells appears to be associated with EGFR-TKI therapeutic efficacy (191, 193). Li et al. showed that EGFR mRNA was expressed in all samples. However, the EGFR expression level increases in malignant tissue compared to normal tissue (70). Based on the research findings, the expression of EGFR and miR-128-b in cancer tissue is expressed differently than in the healthy control tissue; also, the expression profile of miR-128-b is inversely related to the mRNA and protein levels of EGFR. Also, in NSCLC cells, miR-128-b regulated the expression of EGFR, thereby affecting the efficacy of cell therapy. These findings suggest that miR-128-b may have a suppressive function in lung cancer. However, the effectiveness of existing anti-EGFR drugs for cancer diagnosis and treatment is limited, which calls for developing innovative therapeutic techniques to reduce EGFR signal transduction and expression. Inhibition of EGFR expression and EGFR-TKI signaling pathways with miR-128-b may provide a potential drug treatment option in EGFR-mutated NSCLC.

Finally, the effect of DC as an adjuvant therapy for metastatic melanoma is currently being studied in several clinical studies (194, 195). However, cancer can prevent immune recognition by suppressing the maturation and differentiation of DCs, which limits antigen presentation facilitated by DCs (196). In this regard, melanoma is one of the most immunological types of cancer, based on its higher prevalence in immunocompromised patients (197). Efforts to regulate DC communication are essential to define protection against cancer-induced DC abnormalities for effective immunotherapy. Studies show that modulating the p38 MAPK signal transduction pathway affects the growth of immature DCs and T cells, suggesting that p38 seems to be an effective technique for improving DC-mediated cancer immunotherapy (198, 199). Liang and colleagues investigated the effect of the miR-128 expression on p38 in DCs, and the therapeutic benefits (miR-128 and p38) were tested in an animal model bearing melanoma (22). The researchers discovered that the expression of miR-128 was significantly decreased in DCs after stimulation with B16 cell lysate. The miR-128 mimic and inhibitor were delivered to DCs (derived from mouse bone marrow) and then injected into animals with B16 melanoma. These results showed that miR-128 reduces tumorigenesis, increases survival time, and thus has cancer-inhibitory effects (22b). After B16 activation, p38 protein production increased in DCs in their study. A recent report showed that miR-128 mimetics and p38 inhibitors decrease IL-6 and IL-10 secretion while increasing IL-12 levels. Blocking miR-128 had a negative effect on the number of inflammatory cytokines. In conclusion, miR-128 facilitation of anti-tumor (DC-mediated) response in the cancer microenvironment offers a cancer immunotherapy approach against many cancers, such as melanoma.

## 7 Conclusion and future direction

The discovery that 50% of miRNA genes are located in cancer-associated genomic regions or fragile regions, which are commonly increased or decreased during carcinogenesis, emphasizes the relevance of miRNAs in malignancy (200). Cancer is caused by a complex set of gene alterations and is defined by unregulated proliferation, infiltration, and dissemination. Due to their importance in carcinogenesis, miRNAs have been studied as predictive and diagnostic indicators and future therapeutic targets. miR-128 plays an important role in the molecular mechanisms of many types of human cancer. For instance, miR-128 has been shown to suppress the growth of breast cancer by modulating the expression of LINK1 (58). Overexpression of miR-128 in cancer cells inhibited proliferation, migration, and invasion, induced cell apoptosis and suppressed tumor growth.

Conversely, miR-128 has been implicated in miR-128 in carcinogenesis in some cancers. The notion that miR-128 may act as both an anti- and an anti-apoptotic agent suggests that it could be used to treat and develop innovative therapies. Therapeutic approaches based on miR-128 enhancers (miR-128 overexpression) or anti-miRNA oligonucleotides (AMOs) to reduce LNAs (locked nucleic acids) may be studied in an attempt to target malignancy. Consequently, the findings of this analysis will be used to evaluate the possibility of miR-128 as a potential prognostic, diagnostic, and drug target for cancer treatment in the future.

## Author contributions

HSB, LAY, MHL, SP, HJM, and ZHA-q participated in the study design, wrote the draft, and collected the documentation materials. MAJ, RMRP, YFM, FRA, SK, and RM participated in the study design and helped revise the draft. All authors contributed to the article and approved the submitted version.

## References

[1] Siegel RL Miller KD. A Cancer Journal for Clinicians. Cancer Stat (2022) 72:7–33. doi: 10.3322/caac.21708 35020204

[2] Wu W Sun M Zou GM Chen J. MicroRNA and cancer: Current status and prospective. Int J Cancer (2007) 120:953–60. doi: 10.1002/ijc.22454 17163415

[3] Ma L Teruya-Feldstein J Weinberg RA. Tumour invasion and metastasis initiated by microRNA-10b in breast cancer. Nature (2007) 449:682–8. doi: 10.1038/nature06174 17898713

[4] Tavazoie SF Alarcón C Oskarsson T Padua D Wang Q Bos PD. Endogenous human microRNAs that suppress breast cancer metastasis. Nature (2008) 451:147–52. doi: 10.1038/nature06487 PMC2782491 18185580

[5] Costinean S Sandhu SK Pedersen IM Tili E Trotta R Perrotti D. Src homology 2 domain-containing inositol-5-phosphatase and CCAAT enhancer-binding protein beta are targeted by miR-155 in b cells of emicro-MiR-155 transgenic mice. Blood (2009) 114:1374–82. doi: 10.1182/blood-2009-05-220814 PMC2727407 19520806

[6] Su Z Yang Z Xu Y Chen Y Yu Q. MicroRNAs in apoptosis, autophagy and necroptosis. Oncotarget (2015) 6:8474–90. doi: 10.18632/oncotarget.3523 PMC4496162 25893379

[7] Annese T Tamma R De Giorgis M Ribatti D. microRNAs biogenesis, functions and role in tumor angiogenesis. Front Oncol (2020) 10. doi: 10.3389/fonc.2020.581007 PMC7729128 33330058

[8] Jang JH Lee TJ. The role of microRNAs in cell death pathways. Yeungnam Univ J Med (2021) 38:107–17. doi: 10.12701/yujm.2020.00836 PMC8016624 33435638

[9] Di Leva G Garofalo M Croce CM. MicroRNAs in cancer. Annu Rev Pathol (2014) 9:287–314. doi: 10.1146/annurev-pathol-012513-104715 24079833 PMC4009396

[10] Peng Y Croce CM. The role of MicroRNAs in human cancer. Signal Transduct Target Ther (2016) 1:15004. doi: 10.1038/sigtrans.2015.4 29263891 PMC5661652

[11] Tan W Liu B Qu S Liang G Luo W Gong C. MicroRNAs and cancer: Key paradigms in molecular therapy. Oncol Lett (2018) 15:2735–42. 10.3892/ol.2017.7638 PMC5778870 29434998

[12] Wang P Guo X Zong W Song B Liu G He S. MicroRNA-128b suppresses tumor growth and promotes apoptosis by targeting A2bR in gastric cancer. Biochem Biophys Res Commun (2015) 467:798–804. doi: 10.1016/j.bbrc.2015.10.062 26478435

[13] Wang P Guo X Zong W Li Y Liu G Lv Y. PGC-1α/SNAI1 axis regulates tumor growth and metastasis by targeting miR-128b in gastric cancer. (2019) 234:17232–41. 10.1002/jcp.28193 30684287

[14] Fang W Shi C Wang Y Song J Zhang L. microRNA-128-3p inhibits CD4+ regulatory T cells enrichment by targeting interleukin 16 in gastric cancer. (2022) 13:1025–38. doi: 10.1080/21655979.2021.2017566 PMC8805824 34968167

[15] Ching A-S Ahmad-Annuar A. A Perspective on the role of microRNA-128 regulation in mental and behavioral disorders. Frontiers in Cellular Neuroscience (2015) 9:465. 26696825 10.3389/fncel.2015.00465 PMC4677093

[16] Tan CL Plotkin JL Venø MT Von Schimmelmann M Feinberg P Mann S. MicroRNA-128 governs neuronal excitability and motor behavior in mice. Science (2013) 342:1254–8. doi: 10.1126/science.1244193 PMC3932786 24311694

[17] Persengiev SP Kondova II Bontrop RE. The impact of microRNAs on brain aging and neurodegeneration. Curr gerontology geriatrics Res (2012) 2012. doi: 10.1155/2012/359369 PMC3270527 22312330

[18] Weiss GJ Bemis LT Nakajima E Sugita M Birks DK Robinson WA. EGFR regulation by microRNA in lung cancer: correlation with clinical response and survival to gefitinib and EGFR expression in cell lines. Ann Oncol (2008) 19:1053–9. doi: 10.1093/annonc/mdn006 18304967

[19] Kotani A Ha D Hsieh J Rao PK Schotte D Den Boer ML. miR-128b is a potent glucocorticoid sensitizer in MLL-AF4 acute lymphocytic leukemia cells and exerts cooperative effects with miR-221. Blood (2009) 114:4169–78. doi: 10.1182/blood-2008-12-191619 PMC2774553 19749093

[20] Zhu Y Yu F Jiao Y Feng J Tang W Yao H. Reduced miR-128 in breast tumor-initiating cells induces chemotherapeutic resistance *via* bmi-1 and ABCC5. Clin Cancer Res (2011) 17:7105–15. doi: 10.1158/1078-0432.CCR-11-0071 21953503

[21] Adlakha YK Saini N. Brain microRNAs and insights into biological functions and therapeutic potential of brain enriched miRNA-128. Mol Cancer (2014) 13:33. doi: 10.1186/1476-4598-13-33 24555688 PMC3936914

[22] Liang X Shangguan W Zhang M Mei S Wang L Yang R. miR-128 enhances dendritic cell-mediated anti-tumor immunity *via* targeting of p38. Mol Med Rep (2017) 16:1307–13. doi: 10.3892/mmr.2017.6717 PMC5561786 29067466

[23] Zhu Y Yu F Jiao Y Feng J Tang W Yao H. Reduced miR-128 in breast tumor–initiating cells induces chemotherapeutic resistance *via* bmi-1 and ABCC5. Clin Cancer Res (2011) 17:7105–15. doi: 10.1158/1078-0432.CCR-11-0071 21953503

[24] Evangelisti C Florian MC Massimi I Dominici C Giannini G Galardi S. MiR-128 up-regulation inhibits reelin and DCX expression and reduces neuroblastoma cell motility and invasiveness. FASEB J (2009) 23:4276–87. doi: 10.1096/fj.09-134965 19713529

[25] Hu J Cheng Y Li Y Jin Z Pan Y Liu G. microRNA-128 plays a critical role in human non-small cell lung cancer tumourigenesis, angiogenesis and lymphangiogenesis by directly targeting vascular endothelial growth factor-c. Eur J Cancer (2014) 50:2336–50. doi: 10.1016/j.ejca.2014.06.005 25001183

[26] Huang CY Huang XP Zhu JY Chen ZG Li XJ Zhang XH. miR-128-3p suppresses hepatocellular carcinoma proliferation by regulating PIK3R1 and is correlated with the prognosis of HCC patients. Oncol Rep (2015) 33:2889–98. doi: 10.3892/or.2015.3936 25962360

[27] Zhao L Li R Xu S Li Y Zhao P Dong W. Tumor suppressor miR-128-3p inhibits metastasis and epithelial-mesenchymal transition by targeting ZEB1 in esophageal squamous-cell cancer. Acta Biochim Biophys Sin (Shanghai) (2018) 50:171–80. doi: 10.1093/abbs/gmx132 29329360

[28] Li B Chen H Wu N Zhang WJ Shang LX. Deregulation of miR-128 in ovarian cancer promotes cisplatin resistance. Int J Gynecol Cancer (2014) 24:1381–8. doi: 10.1097/IGC.0000000000000252 25248111

[29] Cai J Fang L Huang Y Li R Xu X Hu Z. Simultaneous overactivation of wnt/β-catenin and TGFβ signalling by miR-128-3p confers chemoresistance-associated metastasis in NSCLC. Nat Commun (2017) 8:15870. doi: 10.1038/ncomms15870 28627514 PMC5481840

[30] Bruno IG Karam R Huang L Bhardwaj A Lou CH Shum EY. Identification of a microRNA that activates gene expression by repressing nonsense-mediated RNA decay. Mol Cell (2011) 42:500–10. doi: 10.1016/j.molcel.2011.04.018 PMC3123134 21596314

[31] Karam R Wilkinson M. A conserved microRNA/NMD regulatory circuit controls gene expression. RNA Biol (2012) 9:22–6. doi: 10.4161/rna.9.1.18010 PMC3342942 22258150

[32] Zare M Soleimani M Akbarzadeh A Bakhshandeh B Aghaee-Bakhtiari SH Zarghami N. A novel protocol to differentiate induced pluripotent stem cells by neuronal microRNAs to provide a suitable cellular model. Chem Biol Drug design (2015) 86:232–8. doi: 10.1111/cbdd.12485 25430972

[33] Franzoni E Booker SA Parthasarathy S Rehfeld F Grosser S Srivatsa S. miR-128 regulates neuronal migration, outgrowth and intrinsic excitability *via* the intellectual disability gene Phf6. eLife (2015) 4:e04263. doi: 10.7554/eLife.04263 25556700 PMC4337614

[34] Lin Q Wei W Coelho CM Li X Baker-Andresen D Dudley K. The brain-specific microRNA miR-128b regulates the formation of fear-extinction memory. Nat Neurosci (2011) 14:1115–7. doi: 10.1038/nn.2891 21841775

[35] Shang Q Shen G Chen G Zhang Z Yu X Zhao W. The emerging role of miR-128 in musculoskeletal diseases. J Cell Physiol (2021) 236:4231–43. doi: 10.1002/jcp.30179 33241566

[36] Chen GH Xu CS Zhang J Li Q Cui HH Li XD. Inhibition of miR-128-3p by tongxinluo protects human cardiomyocytes from ischemia/reperfusion injury *via* upregulation of p70s6k1/p-p70s6k1. Front Pharmacol (2017) 8:775. doi: 10.3389/fphar.2017.00775 29163161 PMC5670141

[37] Liu J Wang S Zhang Q Li X Xu S. Selenomethionine alleviates LPS-induced chicken myocardial inflammation by regulating the miR-128-3p-p38 MAPK axis and oxidative stress. Metallomics (2020) 12:54–64. doi: 10.1039/c9mt00216b 31720660

[38] Xie S-J Li J-H Chen H-F Tan Y-Y Liu S-R Zhang Y. Inhibition of the JNK/MAPK signaling pathway by myogenesis-associated miRNAs is required for skeletal muscle development. Cell Death Differentiation (2018) 25:1581–97. doi: 10.1038/s41418-018-0063-1 PMC6143622 29449644

[39] Harding RL Velleman SG. MicroRNA regulation of myogenic satellite cell proliferation and differentiation. Mol Cell Biochem (2016) 412:181–95. doi: 10.1007/s11010-015-2625-6 26715133

[40] Chen C Deng B Qiao M Zheng R Chai J Ding Y. Solexa sequencing identification of conserved and novel microRNAs in backfat of Large white and Chinese meishan pigs. PloS One (2012) 7:e31426. doi: 10.1371/journal.pone.0031426 22355364 PMC3280305

[41] Li HY Xi QY Xiong YY Liu XL Cheng X Shu G. Identification and comparison of microRNAs from skeletal muscle and adipose tissues from two porcine breeds. Anim Genet (2012) 43:704–13. doi: 10.1111/j.1365-2052.2012.02332.x 22497549

[42] Motohashi N Alexander MS Casar JC Kunkel LM. Identification of a novel microRNA that regulates the proliferation and differentiation in muscle side population cells. Stem Cells Dev (2012) 21:3031–43. doi: 10.1089/scd.2011.0721 PMC3475146 22541023

[43] Liew CW Boucher J Cheong JK Vernochet C Koh HJ Mallol C. Ablation of TRIP-Br2, a regulator of fat lipolysis, thermogenesis and oxidative metabolism, prevents diet-induced obesity and insulin resistance. Nat Med (2013) 19:217–26. doi: 10.1038/nm.3056 PMC3567215 23291629

[44] Chen C Deng Y Hu X Ren H Zhu J Fu S. miR-128-3p regulates 3T3-L1 adipogenesis and lipolysis by targeting pparg and Sertad2. J Physiol Biochem (2018) 74:381–93. doi: 10.1007/s13105-018-0625-1 29654510

[45] Na HS Park MH Song YR Kim S Kim HJ Lee JY. Elevated MicroRNA-128 in periodontitis mitigates tumor necrosis factor-α response *via* p38 signaling pathway in macrophages. J Periodontol (2016) 87:e173–182. doi: 10.1902/jop.2016.160033 27240473

[46] Geng L Zhang T Liu W Chen Y. Inhibition of miR-128 abates aβ-mediated cytotoxicity by targeting PPAR-γ *via* NF-κB inactivation in primary mouse cortical neurons and Neuro2a cells. Yonsei Med J (2018) 59:1096–106. doi: 10.3349/ymj.2018.59.9.1096 PMC6192880 30328325

[47] Liu Y Zhang Y Liu P Bai H Li X Xiao J. MicroRNA-128 knockout inhibits the development of alzheimer’s disease by targeting PPARγ in mouse models. Eur J Pharmacol (2019) 843:134–44. doi: 10.1016/j.ejphar.2018.11.004 30412727

[48] Zhang R Liu C Niu Y Jing Y Zhang H Wang J. MicroRNA-128-3p regulates mitomycin c-induced DNA damage response in lung cancer cells through repressing SPTAN1. Oncotarget (2017) 8:58098. doi: 10.18632/oncotarget.12300 28938540 PMC5601636

[49] Zhang M Han W Xu Y Li D Xue Q. Serum miR-128 serves as a potential diagnostic biomarker for alzheimer’s disease. Neuropsychiatr Dis Treat (2021) 17:269–75. doi: 10.2147/NDT.S290925 PMC7853421 33542630

[50] Mi S Lu J Sun M Li Z Zhang H Neilly MB. MicroRNA expression signatures accurately discriminate acute lymphoblastic leukemia from acute myeloid leukemia. Proc Natl Acad Sci U.S.A. (2007) 104:19971–6. 10.1073/pnas.0709313104 PMC2148407 18056805

[51] Li M Fu W Wo L Shu X Liu F Li C. miR-128 and its target genes in tumorigenesis and metastasis. Exp Cell Res (2013) 319:3059–64. doi: 10.1016/j.yexcr.2013.07.031 23958464

[52] Guzman H Sanders K Idica A Bochnakian A Jury D Daugaard I. miR-128 inhibits telomerase activity by targeting TERT mRNA. Oncotarget (2018) 9:13244–53. doi: 10.18632/oncotarget.24284 PMC5862575 29568354

[53] Hamdorf M Idica A Zisoulis DG Gamelin L Martin C Sanders KJ. miR-128 represses L1 retrotransposition by binding directly to L1 RNA. Nat Struct Mol Biol (2015) 22:824–31. doi: 10.1038/nsmb.3090 26367248

[54] Miki Y Nishisho I Horii A Miyoshi Y Utsunomiya J Kinzler KW. Disruption of the APC gene by a retrotransposal insertion of L1 sequence in a colon cancer. Cancer Res (1992) 52:643–5. 1310068

[55] Scott EC Gardner EJ Masood A Chuang NT Vertino PM Devine SE. A hot L1 retrotransposon evades somatic repression and initiates human colorectal cancer. Genome Res (2016) 26:745–55. doi: 10.1101/gr.201814.115 PMC4889970 27197217

[56] Slack FJ Weidhaas JB. MicroRNAs as a potential magic bullet in cancer. Future Oncol (2006) 2:73–82. doi: 10.2217/14796694.2.1.73 16556074

[57] Otmani K Lewalle P. Tumor suppressor miRNA in cancer cells and the tumor microenvironment: Mechanism of deregulation and clinical implications. Front Oncol (2021) 11:708765. doi: 10.3389/fonc.2021.708765 34722255 PMC8554338

[58] Zhao J Li D Fang L. MiR-128-3p suppresses breast cancer cellular progression *via* targeting LIMK1. Biomedicine Pharmacother (2019) 115:108947. doi: 10.1016/j.biopha.2019.108947 31078043

[59] Masri S Liu Z Phung S Wang E Yuan Y-C Chen S. The role of microRNA-128a in regulating TGFbeta signaling in letrozole-resistant breast cancer cells. Breast Cancer Res Treat (2010) 124:89–99. doi: 10.1007/s10549-009-0716-3 20054641 PMC3295576

[60] Breunig C Erdem N Bott A Greiwe JF Reinz E Bernhardt S. TGF β1 regulates HGF-induced cell migration and hepatocyte growth factor receptor MET expression *via* c-ets-1 and miR-128-3p in basal-like breast cancer. Mol Oncol (2018) 12:1447–63. doi: 10.1002/1878-0261.12355 PMC6120235 30004628

[61] Li Y Wang Y Shen X Han X. miR-128 functions as an OncomiR for the downregulation of HIC1 in breast cancer. Front Pharmacol (2019), 1202. doi: 10.3389/fphar.2019.01202 31680974 PMC6811662

[62] Liu S Chen W Hu H Zhang T Wu T Li X. Long noncoding RNA PVT1 promotes breast cancer proliferation and metastasis by binding miR-128-3p and UPF1. Breast Cancer Res (2021) 23:1–13. doi: 10.1186/s13058-021-01491-y 34922601 PMC8684126

[63] Eterno V Zambelli A Villani L Tuscano A Manera S Spitaleri A. AurkA controls self-renewal of breast cancer-initiating cells promoting wnt3a stabilization through suppression of miR-128. Sci Rep (2016) 6:1–13. doi: 10.1038/srep28436 27341528 PMC4920028

[64] Chen Y Wu N Liu L Dong H Liu X. microRNA-128-3p overexpression inhibits breast cancer stem cell characteristics through suppression of wnt signalling pathway by down-regulating NEK2. J Cell Mol Med (2020) 24:7353–69. doi: 10.1111/jcmm.15317 PMC7339185 32558224

[65] Cao D Zhu H Zhao Q Huang J Zhou C He J. MiR-128 suppresses metastatic capacity by targeting metadherin in breast cancer cells. Biol Res (2020) 53:1–13. doi: 10.1186/s40659-020-00311-5 32993809 PMC7526227

[66] Xiao M Lou C Xiao H Yang Y Cai X Li C. MiR-128 regulation of glucose metabolism and cell proliferation in triple-negative breast cancer. J Br Surg (2018) 105:75–85. doi: 10.1002/bjs.10646 29116653

[67] Pan J Zhou C Zhao X He J Tian H Shen W. A two-miRNA signature (miR-33a-5p and miR-128-3p) in whole blood as potential biomarker for early diagnosis of lung cancer. Sci Rep (2018) 8:1–12. doi: 10.1038/s41598-018-35139-3 30420640 PMC6232109

[68] Jiang J Feng X Zhou W Wu Y Yang Y. MiR-128 reverses the gefitinib resistance of the lung cancer stem cells by inhibiting the c-met/PI3K/AKT pathway. Oncotarget (2016) 7:73188–99. doi: 10.18632/oncotarget.12283 PMC5341972 27690301

[69] Cai J Fang L Huang Y Li R Xu X Hu Z. Simultaneous overactivation of wnt/β-catenin and TGFβ signalling by miR-128-3p confers chemoresistance-associated metastasis in NSCLC. Nat Commun (2017) 8:1–19. doi: 10.1038/ncomms15870 28627514 PMC5481840

[70] Li L Wang D. MicroRNA−128−b regulates epidermal growth factor receptor expression in non−small cell lung cancer. Mol Med Rep (2019) 20:4803–10. 10.3892/mmr.2019.10731 PMC6854541 31638205

[71] Donzelli S Fontemaggi G Fazi F Di Agostino S Padula F Biagioni F. MicroRNA-128-2 targets the transcriptional repressor E2F5 enhancing mutant p53 gain of function. Cell Death Differentiation (2012) 19:1038–48. doi: 10.1038/cdd.2011.190 PMC3354056 22193543

[72] Frixa T Sacconi A Cioce M Roscilli G Ferrara FF Aurisicchio L. MicroRNA-128-3p-mediated depletion of drosha promotes lung cancer cell migration. Carcinogenesis (2018) 39:293–304. doi: 10.1093/carcin/bgx134 29236960

[73] Li F Li H Li S Lv B Shi J Yan H. Long non-coding RNA MIAT mediates non-small cell lung cancer development through regulating the miR-128-3p/PELI3 axis. Biochem Genet (2020) 58:867–82. doi: 10.1007/s10528-020-09979-6 32556677

[74] Godlewski J Nowicki MO Bronisz A Williams S Otsuki A Nuovo G. Targeting of the bmi-1 oncogene/stem cell renewal factor by microRNA-128 inhibits glioma proliferation and self-renewal. Cancer Res (2008) 68:9125–30. doi: 10.1158/0008-5472.CAN-08-2629 19010882

[75] Shi Z-M Wang J Yan Z You Y-P Li C-Y Qian X. MiR-128 inhibits tumor growth and angiogenesis by targeting p70S6K1. PloS One (2012) 7:e32709. doi: 10.1371/journal.pone.0032709 22442669 PMC3307714

[76] Ye Y Zhi F Peng Y Yang C. MiR-128 promotes the apoptosis of glioma cells *via* binding to NEK2. Eur Rev Med Pharmacol Sci (2018) 22:8781–8. 10.26355/eurrev_201812_16645 30575919

[77] Fu C Li D Zhang X Liu N Chi G Jin X. LncRNA PVT1 facilitates tumorigenesis and progression of glioma *via* regulation of MiR-128-3p/GREM1 axis and BMP signaling pathway. Neurotherapeutics (2018) 15:1139–57. doi: 10.1007/s13311-018-0649-9 PMC6277294 30120709

[78] Shang C Hong Y Guo Y Liu YH Xue YX. miR-128 regulates the apoptosis and proliferation of glioma cells by targeting RhoE. Oncol Lett (2016) 11:904–8. doi: 10.3892/ol.2015.3927 PMC4727089 26870304

[79] Chen J Wang H Wang J Niu W Deng C Zhou M. LncRNA NEAT1 enhances glioma progression *via* regulating the miR-128-3p/ITGA5 axis. Mol Neurobiol (2021) 58:5163–77. doi: 10.1007/s12035-021-02474-y PMC8497354 34263426

[80] Qu C Yan C Cao W Li F Qu Y Guan K. miR-128-3p contributes to mitochondrial dysfunction and induces apoptosis in glioma cells *via* targeting pyruvate dehydrogenase kinase 1. IUBMB Life (2020) 72:465–75. doi: 10.1002/iub.2212 31828927

[81] Zhao C Guo R Guan F Ma S Li M Wu J. MicroRNA-128-3p enhances the chemosensitivity of temozolomide in glioblastoma by targeting c-met and EMT. Sci Rep (2020) 10:1–12. doi: 10.1038/s41598-020-65331-3 32528036 PMC7289811

[82] Xu J Song J Xiao M Wang C Zhang Q Yuan X. RUNX1 (RUNX family transcription factor 1), a target of microRNA miR-128-3p, promotes temozolomide resistance in glioblastoma multiform by upregulating multidrug resistance-associated protein 1 (MRP1). Bioengineered (2021) 12:11768–81. doi: 10.1080/21655979.2021.2009976 PMC8810036 34895074

[83] She X Yu Z Cui Y Lei Q Wang Z Xu G. miR-128 and miR-149 enhance the chemosensitivity of temozolomide by Rap1B-mediated cytoskeletal remodeling in glioblastoma. Oncol Rep (2014) 32:957–64. doi: 10.3892/or.2014.3318 25017996

[84] Guidi M Muiños-Gimeno M Kagerbauer B Martí E Estivill X Espinosa-Parrilla Y. Overexpression of miR-128 specifically inhibits the truncated isoform of NTRK3 and upregulates BCL2 in SH-SY5Y neuroblastoma cells. BMC Mol Biol (2010) 11:1–17. doi: 10.1186/1471-2199-11-95 21143953 PMC3019150

[85] Bao J Zhang S Meng Q Qin T. SNHG16 silencing inhibits neuroblastoma progression by downregulating HOXA7 *via* sponging miR-128-3p. Neurochemical Res (2020) 45:825–36. doi: 10.1007/s11064-020-02955-x 31919621

[86] Cao X-Z Bin H Zang Z-N. MiR-128 suppresses the growth of thyroid carcinoma by negatively regulating SPHK1. Biomedicine Pharmacother (2019) 109:1960–6. doi: 10.1016/j.biopha.2018.08.052 30551451

[87] Chen J Zhao D Meng Q. Knockdown of HCP5 exerts tumor-suppressive functions by up-regulating tumor suppressor miR-128-3p in anaplastic thyroid cancer. Biomedicine Pharmacother (2019) 116:108966. doi: 10.1016/j.biopha.2019.108966 31102936

[88] Zhang C Xie L Liang H Cui Y. LncRNA MIAT facilitates osteosarcoma progression by regulating mir-128-3p/VEGFC axis. IUBMB Life (2019) 71:845–53. doi: 10.1002/iub.2001 30629798

[89] Liu Q Ran R Song M Li X Wu Z Dai G. LncRNA HCP5 acts as a miR-128-3p sponge to promote the progression of multiple myeloma through activating wnt/β-catenin/cyclin D1 signaling *via* PLAGL2. Cell Biol Toxicol (2021), 1–15. 10.1007/s10565-021-09628-7 34331612

[90] Wan G-L Chen H Zhou L Huang J-M. Overexpressed miR-128a inhibits the proliferation of laryngeal cancer cells. Transl Cancer Res (2018) 7:901–11. doi: 10.21037/tcr.2018.06.13

[91] Hauser B Zhao Y Pang X Ling Z Myers E Wang P. Functions of MiRNA-128 on the regulation of head and neck squamous cell carcinoma growth and apoptosis. PloS One (2015) 10:e0116321. doi: 10.1371/journal.pone.0116321 25764126 PMC4357443

[92] Zhang Y Chao T Li R Liu W Chen Y Yan X. MicroRNA-128 inhibits glioma cells proliferation by targeting transcription factor E2F3a. J Mol Med (2009) 87:43–51. doi: 10.1007/s00109-008-0403-6 18810376

[93] Khan AP Poisson LM Bhat VB Fermin D Zhao R Kalyana-Sundaram S. Quantitative proteomic profiling of prostate cancer reveals a role for miR-128 in prostate cancer. Mol Cell Proteomics (2010) 9:298–312. doi: 10.1074/mcp.M900159-MCP200 19955085 PMC2830841

[94] Kotani A Ha D Schotte D Den Boer ML Armstrong SA Lodish HF. A novel mutation in the miR-128b gene reduces miRNA processing and leads to glucocorticoid resistance of MLL-AF4 acute lymphocytic leukemia cells. Cell Cycle (2010) 9:1037–42. doi: 10.4161/cc.9.6.11011 PMC3096720 20237425

[95] Cao D Zhu H Zhao Q Huang J Zhou C He J. MiR-128 suppresses metastatic capacity by targeting metadherin in breast cancer cells. Biol Res (2020) 53:43–3. doi: 10.1186/s40659-020-00311-5 PMC7526227 32993809

[96] Li XQ Lu JT Tan CC Wang QS Feng YM. RUNX2 promotes breast cancer bone metastasis by increasing integrin α5-mediated colonization. Cancer Lett (2016) 380:78–86. doi: 10.1016/j.canlet.2016.06.007 27317874

[97] Iriondo O Liu Y Lee G Elhodaky M. TAK1 mediates microenvironment-triggered autocrine signals and promotes triple-negative breast cancer lung metastasis. (2018) 9:1994. doi: 10.1038/s41467-018-04460-w PMC5959931 29777109

[98] Mandujano-Tinoco EA García-Venzor A Melendez-Zajgla J Maldonado V. New emerging roles of microRNAs in breast cancer. Breast Cancer Res Treat (2018) 171:247–59. doi: 10.1007/s10549-018-4850-7 29948402

[99] Piasecka D Braun M Kordek R Sadej R Romanska H. MicroRNAs in regulation of triple-negative breast cancer progression. J Cancer Res Clin Oncol (2018) 144:1401–11. doi: 10.1007/s00432-018-2689-2 PMC6061037 29923083

[100] Ma L. MicroRNA and metastasis. Adv Cancer Res (2016) 132:165–207. doi: 10.1016/bs.acr.2016.07.004 27613133

[101] Xiao M Lou C Xiao H Yang Y Cai X Li C. MiR-128 regulation of glucose metabolism and cell proliferation in triple-negative breast cancer. Br J Surg (2017) 105:75–85. doi: 10.1002/bjs.10646 29116653

[102] Anastas JN Moon RT. WNT signalling pathways as therapeutic targets in cancer. Nat Rev Cancer (2013) 13:11–26. doi: 10.1038/nrc3419 23258168

[103] Tan Z Zheng H Liu X Zhang W Zhu J Wu G. MicroRNA-1229 overexpression promotes cell proliferation and tumorigenicity and activates wnt/β-catenin signaling in breast cancer. Oncotarget (2016) 7:24076–87. doi: 10.18632/oncotarget.8119 PMC5029685 26992223

[104] Tang X Wang Z Lei T Zhou W Chang S Li D. Importance of protein flexibility on molecular recognition: modeling binding mechanisms of aminopyrazine inhibitors to Nek2. Phys Chem Chem Phys (2018) 20:5591–605. doi: 10.1039/C7CP07588J 29270587

[105] Lee M Oprea-Ilies G Saavedra HI. Silencing of E2F3 suppresses tumor growth of Her2+ breast cancer cells by restricting mitosis. Oncotarget (2015) 6:37316–34. doi: 10.18632/oncotarget.5686 PMC4741932 26512919

[106] Nuncia-Cantarero M Martinez-Canales S Andrés-Pretel F Santpere G Ocaña A. Functional transcriptomic annotation and protein-protein interaction network analysis identify NEK2, BIRC5, and TOP2A as potential targets in obese patients with luminal a breast cancer. (2018) 168:613–23. doi: 10.1007/s10549-017-4652-3 PMC5842257 29330624

[107] Turashvili G Lightbody ED Tyryshkin K Sengupta SK Elliott BE Madarnas Y. Novel prognostic and predictive microRNA targets for triple-negative breast cancer. FASEB J (2018), fj201800120R. doi: 10.1096/fj.201800120R 29812973

[108] Luo J Solimini NL Elledge SJ. Principles of cancer therapy: oncogene and non-oncogene addiction. Cell (2009) 136:823–37. doi: 10.1016/j.cell.2009.02.024 PMC2894612 19269363

[109] Pinte S Stankovic-Valentin N Deltour S Rood BR Guérardel C Leprince D. The tumor suppressor gene HIC1 (hypermethylated in cancer 1) is a sequence-specific transcriptional repressor: definition of its consensus binding sequence and analysis of its DNA binding and repressive properties. J Biol Chem (2004) 279:38313–24. doi: 10.1074/jbc.M401610200 15231840

[110] Chen WY Wang DH Yen RC Luo J Gu W Baylin SB. Tumor suppressor HIC1 directly regulates SIRT1 to modulate p53-dependent DNA-damage responses. Cell (2005) 123:437–48. doi: 10.1016/j.cell.2005.08.011 16269335

[111] Morton RA Watkins JJ Bova SG Wales MM Baylin SB Isaacs WB. Hypermethylation of chromosome 17P locust D17S5 in human prostate tissue. J Urol (1996) 156:512–6. doi: 10.1016/S0022-5347(01)65916-0 8683727

[112] Zhang W Zeng X Briggs KJ Beaty R Simons B Chiu Yen R. A potential tumor suppressor role for Hic1 in breast cancer through transcriptional repression of ephrin-A1. Oncogene (2010) 29:2467–76. doi: 10.1038/onc.2010.12 PMC3025282 20154726

[113] Jin F Wang Y Li M Zhu Y Liang H Wang C. MiR-26 enhances chemosensitivity and promotes apoptosis of hepatocellular carcinoma cells through inhibiting autophagy. Cell Death Dis (2018) 8:e2540–0. 10.1038/cddis.2016.461 PMC5386370 28079894

[114] Yang J Li J Le Y Zhou C Zhang S Gong Z. PFKL/miR-128 axis regulates glycolysis by inhibiting AKT phosphorylation and predicts poor survival in lung cancer. Am J Cancer Res (2016) 6:473–85. PMC4859674 27186417

[115] Pan J Zhou C Zhao X He J Tian H Shen W. A two-miRNA signature (miR-33a-5p and miR-128-3p) in whole blood as potential biomarker for early diagnosis of lung cancer. Sci Rep (2018) 8:16699. doi: 10.1038/s41598-018-35139-3 30420640 PMC6232109

[116] Zhang R Liu C Niu Y Jing Y Zhang H Wang J. MicroRNA-128-3p regulates mitomycin c-induced DNA damage response in lung cancer cells through repressing SPTAN1. Oncotarget (2016) 8:58098–107. doi: 10.18632/oncotarget.12300 PMC5601636 28938540

[117] Gupta-Abramson V Troxel AB Nellore A Puttaswamy K Redlinger M Ransone K. Phase II trial of sorafenib in advanced thyroid cancer. J Clin Oncol (2008) 26:4714. doi: 10.1200/JCO.2008.16.3279 18541894 PMC2653134

[118] Griffith OL Melck A Jones SJ Wiseman SM. Meta-analysis and meta-review of thyroid cancer gene expression profiling studies identifies important diagnostic biomarkers. J Clin Oncol (2006) 24:5043–51. doi: 10.1200/JCO.2006.06.7330 17075124

[119] Xing M. Molecular pathogenesis and mechanisms of thyroid cancer. Nat Rev Cancer (2013) 13:184–99. doi: 10.1038/nrc3431 PMC3791171 23429735

[120] Shida D Takabe K Kapitonov D Milstien S Spiegel S. Targeting SphK1 as a new strategy against cancer. Curr Drug Targets (2008) 9:662–73. doi: 10.2174/138945008785132402 PMC2674575 18691013

[121] Pasieka JL. Anaplastic thyroid cancer. Curr Opin Oncol (2003) 15:78–83. doi: 10.1097/00001622-200301000-00012 12490766

[122] Ranganath R Shah MA Shah AR. Anaplastic thyroid cancer. *Current opinion in endocrinology, diabetes and obesity* . (2015) 22:387–91. 10.1097/MED.0000000000000189 26313900

[123] Smallridge R Copland J. Anaplastic thyroid carcinoma: pathogenesis and emerging therapies. Clin Oncol (2010) 22:486–97. doi: 10.1016/j.clon.2010.03.013 PMC3905320 20418080

[124] Liang L Xu J Wang M Xu G Zhang N Wang G. LncRNA HCP5 promotes follicular thyroid carcinoma progression *via* miRNAs sponge. Cell Death Dis (2018) 9:1–13. doi: 10.1038/s41419-018-0382-7 29515098 PMC5841368

[125] Cohen Y Xing M Mambo E Guo Z Wu G Trink B. BRAF mutation in papillary thyroid carcinoma. J Natl Cancer Inst (2003) 95:625–7. doi: 10.1093/jnci/95.8.625 12697856

[126] Jemal A Siegel R Ward E Murray T Xu J Thun MJ. Cancer statistic. CA Cancer J Clin (2007) 57:43–66. doi: 10.3322/canjclin.57.1.43 17237035

[127] Forastiere A Koch W Trotti A Sidransky D. Head and neck cancer. N Engl J Med (2001) 345:1890–900. doi: 10.1056/NEJMra001375 11756581

[128] Lippman SM Sudbø J Hong WK. Oral cancer prevention and the evolution of molecular-targeted drug development. J Clin Oncol (2005) 23:346–56. doi: 10.1200/JCO.2005.09.128 15637397

[129] Cui JG Zhao Y Sethi P Li YY Mahta A Culicchia F. Micro-RNA-128 (miRNA-128) down-regulation in glioblastoma targets ARP5 (ANGPTL6), bmi-1 and E2F-3a, key regulators of brain cell proliferation. J Neurooncol (2010) 98:297–304. doi: 10.1007/s11060-009-0077-0 19941032

[130] Chen H Zhou L Dou T Wan G Tang H Tian J. BMI1’S maintenance of the proliferative capacity of laryngeal cancer stem cells. Head Neck (2011) 33:1115–25. doi: 10.1002/hed.21576 21755556

[131] Chen H Zhou L Wan G Dou T Tian J. BMI1 promotes the progression of laryngeal squamous cell carcinoma. Oral Oncol (2011) 47:472–81. doi: 10.1016/j.oraloncology.2011.03.016 21482478

[132] Jemal A Bray F Center MM Ferlay J Ward E Forman D. Global cancer statistics. CA Cancer J Clin (2011) 61:69–90. doi: 10.3322/caac.20107 21296855

[133] Hameed M Mandelker D. Tumor syndromes predisposing to osteosarcoma. Adv Anat Pathol (2018) 25:217–22. doi: 10.1097/PAP.0000000000000190 PMC6688172 29668499

[134] Harrison DJ Geller DS Gill JD Lewis VO Gorlick R. Current and future therapeutic approaches for osteosarcoma. Expert Rev Anticancer Ther (2018) 18:39–50. doi: 10.1080/14737140.2018.1413939 29210294

[135] Wang J Cao L Wu J Wang Q. Long non-coding RNA SNHG1 regulates NOB1 expression by sponging miR-326 and promotes tumorigenesis in osteosarcoma. Int J Oncol (2018) 52:77–88. 29115574 10.3892/ijo.2017.4187 PMC5743365

[136] Chan JJ Tay Y. Noncoding RNA: RNA regulatory networks in cancer. Int J Mol Sci (2018) 19:1310. doi: 10.3390/ijms19051310 29702599 PMC5983611

[137] Zhou J He Z Guo L Zeng J Liang P Ren L. MiR-128-3p directly targets VEGFC/VEGFR3 to modulate the proliferation of lymphatic endothelial cells through Ca(2+) signaling. Int J Biochem Cell Biol (2018) 102:51–8. doi: 10.1016/j.biocel.2018.05.006 29777777

[138] Davis FG Mccarthy BJ. Current epidemiological trends and surveillance issues in brain tumors. Expert Rev Anticancer Ther (2001) 1:395–401. doi: 10.1586/14737140.1.3.395 12113106

[139] Ciafrè SA Galardi S Mangiola A Ferracin M Liu CG Sabatino G. Extensive modulation of a set of microRNAs in primary glioblastoma. Biochem Biophys Res Commun (2005) 334:1351–8. doi: 10.1016/j.bbrc.2005.07.030 16039986

[140] Kefas B Godlewski J Comeau L Li Y Abounader R Hawkinson M. microRNA-7 inhibits the epidermal growth factor receptor and the akt pathway and is down-regulated in glioblastoma. Cancer Res (2008) 68:3566–72. doi: 10.1158/0008-5472.CAN-07-6639 18483236

[141] Kim H Huang W Jiang X Pennicooke B Park PJ Johnson MD. Integrative genome analysis reveals an oncomir/oncogene cluster regulating glioblastoma survivorship. Proc Natl Acad Sci U.S.A. (2010) 107:2183–8. 10.1073/pnas.0909896107 PMC2836668 20080666

[142] Zencak D Lingbeek M Kostic C Tekaya M Tanger E Hornfeld D. Bmi1 loss produces an increase in astroglial cells and a decrease in neural stem cell population and proliferation. J Neurosci (2005) 25:5774–83. doi: 10.1523/JNEUROSCI.3452-04.2005 PMC6724881 15958744

[143] Sparmann A Van Lohuizen M. Polycomb silencers control cell fate, development and cancer. Nat Rev Cancer (2006) 6:846–56. doi: 10.1038/nrc1991 17060944

[144] Bruggeman SW Hulsman D Tanger E Buckle T Blom M Zevenhoven J. Bmi1 controls tumor development in an Ink4a/Arf-independent manner in a mouse model for glioma. Cancer Cell (2007) 12:328–41. doi: 10.1016/j.ccr.2007.08.032 17936558

[145] Qu C Yan C Cao W Li F Qu Y Guan K. miR-128-3p contributes to mitochondrial dysfunction and induces apoptosis in glioma cells *via* targeting pyruvate dehydrogenase kinase. (2020) 1. 72:465–75. doi: 10.1002/iub.2212 31828927

[146] Abedalthagafi M Barakeh D Foshay KM. Immunogenetics of glioblastoma: the future of personalized patient management. NPJ Precis Oncol (2018) 2:27. doi: 10.1038/s41698-018-0070-1 30534602 PMC6279755

[147] Sumazin P Yang X Chiu HS Chung WJ Iyer A Llobet-Navas D. An extensive microRNA-mediated network of RNA-RNA interactions regulates established oncogenic pathways in glioblastoma. Cell (2011) 147:370–81. doi: 10.1016/j.cell.2011.09.041 PMC3214599 22000015

[148] Teng H Wang P Xue Y Liu X Ma J Cai H. Role of HCP5-miR-139-RUNX1 feedback loop in regulating malignant behavior of glioma cells. Mol Ther (2016) 24:1806–22. doi: 10.1038/mt.2016.103 PMC5112034 27434586

[149] Zhao K Cui X Wang Q Fang C Tan Y Wang Y. ). RUNX1 contributes to the mesenchymal subtype of glioblastoma in a TGFβ pathway-dependent manner. (2019) 10:877. 10.1038/s41419-019-2108-x PMC6872557 31754093

[150] Li W Ma Q Liu Q Yan P Wang X Jia X. Circ-VPS18 knockdown enhances TMZ sensitivity and inhibits glioma progression by MiR-370/RUNX1 axis. (2021) 71:1234–44. doi: 10.1007/s12031-020-01749-8 33188501

[151] Schinkel AH. The roles of p-glycoprotein and MRP1 in the blood-brain and blood-cerebrospinal fluid barriers. Adv Exp Med Biol (2001) 500:365–72. doi: 10.1007/978-1-4615-0667-6_60 11764971

[152] Munoz M Henderson M Haber M Norris M. Role of the MRP1/ABCC1 multidrug transporter protein in cancer. IUBMB Life (2007) 59:752–7. doi: 10.1080/15216540701736285 18085475

[153] Look AT. Oncogenic transcription factors in the human acute leukemias. Science (1997) 278:1059–64. doi: 10.1126/science.278.5340.1059 9353180

[154] Rowley JD. Molecular genetics in acute leukemia. Leukemia (2000) 14:513–7. doi: 10.1038/sj.leu.2401600 10720153

[155] Pui CH Schrappe M Ribeiro RC Niemeyer CM. Childhood and adolescent lymphoid and myeloid leukemia. Hematol Am Soc Hematol Educ Program (2004), 118–45. doi: 10.1182/asheducation-2004.1.118 15561680

[156] Randolph TR. Advances in acute lymphoblastic leukemia. Clin Lab Sci (2004) 17:235–45. 15559730

[157] Löwenberg B Downing JR Burnett A. Acute myeloid leukemia. N Engl J Med (1999) 341:1051–62. doi: 10.1056/NEJM199909303411407 10502596

[158] Pui CH Chessells JM Camitta B Baruchel A Biondi A Boyett JM. Clinical heterogeneity in childhood acute lymphoblastic leukemia with 11q23 rearrangements. Leukemia (2003) 17:700–6. doi: 10.1038/sj.leu.2402883 12682627

[159] Lapidot T Sirard C Vormoor J Murdoch B Hoang T Caceres-Cortes J. A cell initiating human acute myeloid leukaemia after transplantation into SCID mice. Nature (1994) 367:645–8. doi: 10.1038/367645a0 7509044

[160] Al-Hajj M Wicha MS Benito-Hernandez A Morrison SJ Clarke MF. Prospective identification of tumorigenic breast cancer cells. Proc Natl Acad Sci (2003) 100:3983–8. doi: 10.1073/pnas.0530291100 PMC153034 12629218

[161] Singh SK Clarke ID Terasaki M Bonn VE Hawkins C Squire J. Identification of a cancer stem cell in human brain tumors. Cancer Res (2003) 63:5821–8. 14522905

[162] Galli R Binda E Orfanelli U Cipelletti B Gritti A De Vitis S. Isolation and characterization of tumorigenic, stem-like neural precursors from human glioblastoma. Cancer Res (2004) 64:7011–21. doi: 10.1158/0008-5472.CAN-04-1364 15466194

[163] Ponti D Costa A Zaffaroni N Pratesi G Petrangolini G Coradini D. Isolation and *in vitro* propagation of tumorigenic breast cancer cells with stem/progenitor cell properties. Cancer Res (2005) 65:5506–11. doi: 10.1158/0008-5472.CAN-05-0626 15994920

[164] Patrawala L Calhoun T Schneider-Broussard R Li H Bhatia B Tang S. Highly purified CD44+ prostate cancer cells from xenograft human tumors are enriched in tumorigenic and metastatic progenitor cells. Oncogene (2006) 25:1696–708. doi: 10.1038/sj.onc.1209327 16449977

[165] Vescovi AL Galli R Reynolds BA. Brain tumour stem cells. Nat Rev Cancer (2006) 6:425–36. doi: 10.1038/nrc1889 16723989

[166] Li C Heidt DG Dalerba P Burant CF Zhang L Adsay V. Identification of pancreatic cancer stem cells. Cancer Res (2007) 67:1030–7. doi: 10.1158/0008-5472.CAN-06-2030 17283135

[167] Ma S Chan KW Hu L Lee TK Wo JY Ng IO. Identification and characterization of tumorigenic liver cancer stem/progenitor cells. Gastroenterology (2007) 132:2542–56. doi: 10.1053/j.gastro.2007.04.025 17570225

[168] O’brien CA Pollett A Gallinger S Dick JE. A human colon cancer cell capable of initiating tumour growth in immunodeficient mice. Nature (2007) 445:106–10. doi: 10.1038/nature05372 17122772

[169] Ricci-Vitiani L Lombardi DG Pilozzi E Biffoni M Todaro M Peschle C. Identification and expansion of human colon-cancer-initiating cells. Nature (2007) 445:111–5. doi: 10.1038/nature05384 17122771

[170] Costello RT Mallet F Gaugler B Sainty D Arnoulet C Gastaut JA. Human acute myeloid leukemia CD34+/CD38- progenitor cells have decreased sensitivity to chemotherapy and fas-induced apoptosis, reduced immunogenicity, and impaired dendritic cell transformation capacities. Cancer Res (2000) 60:4403–11. 10969785

[171] Guzman ML Swiderski CF Howard DS Grimes BA Rossi RM Szilvassy SJ. Preferential induction of apoptosis for primary human leukemic stem cells. Proc Natl Acad Sci U.S.A. (2002) 99:16220–5. 10.1073/pnas.252462599 PMC138592 12451177

[172] Dalerba P Cho RW Clarke MF. Cancer stem cells: models and concepts. Annu Rev Med (2007) 58:267–84. doi: 10.1146/annurev.med.58.062105.204854 17002552

[173] Lagos-Quintana M Rauhut R Lendeckel W Tuschl T. Identification of novel genes coding for small expressed RNAs. Science (2001) 294:853–8. doi: 10.1126/science.1064921 11679670

[174] Zhao C Guo R Guan F Ma S Li M Wu J. MicroRNA-128-3p enhances the chemosensitivity of temozolomide in glioblastoma by targeting c-met and EMT. Sci Rep (2020) 10:9471–1. doi: 10.1038/s41598-020-65331-3 PMC7289811 32528036

[175] She X Yu Z Cui Y Lei Q Wang Z Xu G. miR-128 and miR-149 enhance the chemosensitivity of temozolomide by Rap1B-mediated cytoskeletal remodeling in glioblastoma. Oncol Rep (2014) 32:957–64. doi: 10.3892/or.2014.3318 25017996

[176] Dragomir M Chen B Fu X Calin GA. Key questions about the checkpoint blockade-are microRNAs an answer? Cancer Biol Med (2018) 15:103–15. doi: 10.20892/j.issn.2095-3941.2018.0006 PMC5994554 29951335

[177] Cortez MA Anfossi S Ramapriyan R Menon H Atalar SC Aliru M. Role of miRNAs in immune responses and immunotherapy in cancer. Genes Chromosomes Cancer (2019) 58:244–53. doi: 10.1002/gcc.22725 PMC6368474 30578699

[178] Mehta A Baltimore D. MicroRNAs as regulatory elements in immune system logic. Nat Rev Immunol (2016) 16:279–94. doi: 10.1038/nri.2016.40 27121651

[179] Xing Y Wang Z Lu Z Xia J Xie Z Jiao M. MicroRNAs: immune modulators in cancer immunotherapy. Immunother Adv (2021) 1. doi: 10.1093/immadv/ltab006 PMC9327120 35919742

[180] Lee HM Nguyen DT Lu LF. Progress and challenge of microRNA research in immunity. Front Genet (2014) 5:178. doi: 10.3389/fgene.2014.00178 24971086 PMC4053854

[181] Paladini L Fabris L Bottai G Raschioni C Calin GA Santarpia L. Targeting microRNAs as key modulators of tumor immune response. J Exp Clin Cancer Res (2016) 35:103. doi: 10.1186/s13046-016-0375-2 27349385 PMC4924278

[182] Zamarron BF Chen W. Dual roles of immune cells and their factors in cancer development and progression. Int J Biol Sci (2011) 7:651–8. doi: 10.7150/ijbs.7.651 PMC3107473 21647333

[183] Yin Y Cai X Chen X Liang H Zhang Y Li J. Tumor-secreted miR-214 induces regulatory T cells: a major link between immune evasion and tumor growth. Cell Res (2014) 24:1164–80. doi: 10.1038/cr.2014.121 PMC4185347 25223704

[184] Zhang L Lei J Fang ZL Xiong JP. MiR-128b is down-regulated in gastric cancer and negatively regulates tumour cell viability by targeting PDK1/Akt/NF-κB axis. J Biosci (2016) 41:77–85. doi: 10.1007/s12038-016-9586-0 26949090

[185] Wang X Xin S Wang Y Ju D Wu Q Qiu Y. MicroRNA-146a-5p enhances T helper 17 cell differentiation *via* decreasing a disintegrin and metalloprotease 17 level in primary sjögren’s syndrome. Bioengineered (2021) 12:310–24. doi: 10.1080/21655979.2020.1870321 PMC8806215 33446013

[186] Zheng T Han W Wang A Wang Y. Functional mechanism of hsa-miR-128-3p in epithelial-mesenchymal transition of pancreatic cancer cells *via* ZEB1 regulation. PeerJ (2022) 10:e12802. doi: 10.7717/peerj.12802 35186455 PMC8818272

[187] Ahn HK Choi YL Han JH Ahn YC Kim K Kim J. Epidermal growth factor receptor mutation and treatment outcome of mediastinoscopic N2 positive non-small cell lung cancer patients treated with neoadjuvant chemoradiotherapy followed by surgery. Lung Cancer (2013) 79:300–6. doi: 10.1016/j.lungcan.2012.11.010 23261144

[188] Tanaka K Hida T Oya Y Oguri T Yoshida T Shimizu J. EGFR mutation impact on definitive concurrent chemoradiation therapy for inoperable stage III adenocarcinoma. J Thorac Oncol (2015) 10:1720–5. doi: 10.1097/JTO.0000000000000675 26743855

[189] Dahabreh IJ Linardou H Kosmidis P Bafaloukos D Murray S. EGFR gene copy number as a predictive biomarker for patients receiving tyrosine kinase inhibitor treatment: a systematic review and meta-analysis in non-small-cell lung cancer. Ann Oncol (2011) 22:545–52. doi: 10.1093/annonc/mdq432 20826716

[190] Pirker R. What is the best strategy for targeting EGF receptors in non-small-cell lung cancer? Future Oncol (2015) 11:153–67. doi: 10.2217/fon.14.178 25572790

[191] Duan X Shi J. [Advance in microRNAs and EGFR-TKIs secondary resistance research in non-small cell lung cancer]. Zhongguo Fei Ai Za Zhi (2014) 17:860–4. 10.3779/j.issn.1009-3419.2014.12.07 PMC6000411 25539612

[192] Zhao N Zhang XC Yan HH Yang JJ Wu YL. Efficacy of epidermal growth factor receptor inhibitors versus chemotherapy as second-line treatment in advanced non-small-cell lung cancer with wild-type EGFR: a meta-analysis of randomized controlled clinical trials. Lung Cancer (2014) 85:66–73. doi: 10.1016/j.lungcan.2014.03.026 24780111

[193] Wang S Su X Bai H Zhao J Duan J An T. Identification of plasma microRNA profiles for primary resistance to EGFR-TKIs in advanced non-small cell lung cancer (NSCLC) patients with EGFR activating mutation. J Hematol Oncol (2015) 8:127. doi: 10.1186/s13045-015-0210-9 26563758 PMC4643502

[194] Mackensen A Herbst B Chen JL Köhler G Noppen C Herr W. Phase I study in melanoma patients of a vaccine with peptide-pulsed dendritic cells generated *in vitro* from CD34(+) hematopoietic progenitor cells. Int J Cancer (2000) 86:385–92. doi: 10.1002/(SICI)1097-0215(20000501)86:3<385::AID-IJC13>3.0.CO;2-T 10760827

[195] Bol KF Aarntzen EH Hout FE Schreibelt G Creemers JH Lesterhuis WJ. Favorable overall survival in stage III melanoma patients after adjuvant dendritic cell vaccination. Oncoimmunology (2016) 5:e1057673. doi: 10.1080/2162402X.2015.1057673 26942068 PMC4760342

[196] Gabrilovich D. Mechanisms and functional significance of tumour-induced dendritic-cell defects. Nat Rev Immunol (2004) 4:941–52. doi: 10.1038/nri1498 15573129

[197] Ralli M Botticelli A. ). immunotherapy in the treatment of metastatic melanoma: Current knowledge and future directions. (2020) 2020:9235638. doi: 10.1155/2020/9235638 PMC7338969 32671117

[198] Xie J Qian J Yang J Wang S Freeman ME 3rd Yi Q. Critical roles of Raf/MEK/ERK and PI3K/AKT signaling and inactivation of p38 MAP kinase in the differentiation and survival of monocyte-derived immature dendritic cells. Exp Hematol (2005) 33:564–72. doi: 10.1016/j.exphem.2005.03.001 15850834

[199] Wang S Hong S Yang J Qian J Zhang X Shpall E. Optimizing immunotherapy in multiple myeloma: Restoring the function of patients’ monocyte-derived dendritic cells by inhibiting p38 or activating MEK/ERK MAPK and neutralizing interleukin-6 in progenitor cells. Blood (2006) 108:4071–7. doi: 10.1182/blood-2006-04-016980 PMC1895445 16917008

[200] Condrat CE Thompson DC Barbu MG Bugnar OL Boboc A Cretoiu D. miRNAs as biomarkers in disease: Latest findings regarding their role in diagnosis and prognosis. Cells (2020) 9. doi: 10.3390/cells9020276 PMC7072450 31979244

